# DNA damage signalling from the placenta to foetal blood as a potential mechanism for childhood leukaemia initiation

**DOI:** 10.1038/s41598-019-39552-0

**Published:** 2019-03-13

**Authors:** Els Mansell, Nahid Zareian, Camille Malouf, Chrysa Kapeni, Natalie Brown, Christophe Badie, Duncan Baird, Jon Lane, Katrin Ottersbach, Allison Blair, C. Patrick Case

**Affiliations:** 1 School of Clinical Science, University of Bristol, Learning and Research Centre, Southmead Hospital, Bristol, UK; 2 MRC Centre for Regenerative Medicine, SCRM Building, The University of Edinburgh, Edinburgh Bioquarter 5 Little France Drive, Edinburgh, UK; 3 Cancer Mecanisms and Biomarkers, Department of Radiation Effects, Public Health England’s Centre for Radiation, Chemical and Environmental Hazards (CRCE), Chilton, Didcot, Oxon UK; 4 Division of Cancer & Genetics, School of Medicine, Cardiff University, Cardiff, UK; 5 School of Cellular and Molecular Medicine, University of Bristol, Bristol, UK; 6 School of Biochemistry, University of Bristol, Bristol, UK; 7 Bristol Institute for Transfusion Sciences, NHS Blood and Transplant, Filton, UK

## Abstract

For many diseases with a foetal origin, the cause for the disease initiation remains unknown. Common childhood acute leukaemia is thought to be caused by two hits, the first *in utero* and the second in childhood in response to infection. The mechanism for the initial DNA damaging event are unknown. Here we have used *in vitro*, *ex vivo* and *in vivo* models to show that a placental barrier will respond to agents that are suspected of initiating childhood leukaemia by releasing factors that cause DNA damage in cord blood and bone marrow cells, including stem cells. We show that DNA damage caused by *in utero* exposure can reappear postnatally after an immune challenge. Furthermore, both foetal and postnatal DNA damage are prevented by prenatal exposure of the placenta to a mitochondrially-targeted antioxidant. We conclude that the placenta might contribute to the first hit towards leukaemia initiation by bystander-like signalling to foetal haematopoietic cells.

## Introduction

For an increasing number of adult and childhood diseases, the evidence points to a foetal origin^1–3^. In the case of common childhood acute leukaemia, the ‘two-hit hypothesis’ proposes that a pre-leukaemic state is established *in utero*, characterised by DNA damage to haematopoietic stem or progenitor cells in the form of chromosomal translocations or aneuploidy^4–6^. This initiating event predisposes to overt leukaemia, following a second genetic hit during childhood that has been linked to an aberrant response following (delayed encounter of) common infection^7–9^. Childhood leukaemia is the most common paediatric malignancy, and the incidence is steadily rising in developed countries^10,11^. The *in utero* origin of common childhood acute lymphocytic leukaemia (cALL)^12–16^, and to some extent acute myeloid leukaemia (AML), has been well documented using twin studies and retrospective scrutiny of patients’ neonatal blood spots^16–18^. However, the causes for the DNA damage in foetal haematopoietic cells required for the initiating event, remain enigmatic.

Certain epidemiological studies report a link between maternal exposure during pregnancy and an increased risk of childhood leukaemia in the offspring (summarised in Table 1).

**Table 1:** Summarised overview of maternal exposures that are investigated, and the strength of their association, with leukaemia.

Maternal exposure agent	Strength of link	References
Chromium	~	^58– 60^
Hypoxia	~	^61, 62^
Infection	+/−	^63– 70^
DNA topoisomerase II inhibitors	+/−	^71– 75^
Pesticides	+	^30, 76– 80^
Benzene	++	^81– 84^
Ionising radiation	+++	^30– 33, 82^

^*^~Infrequently reported and mixed results; +/−frequently investigated and mixed results, +frequently positively associated; ++frequent and consistent evidence reported, +++proven association.

Although these epidemiological studies are informative, they are troubled by contradictory results, low study numbers, recall bias due to their retrospective nature and socio-economic, environmental and genetic factors that influence the results. Experimental evidence is required to bridge the gap between these epidemiological studies and clinical observations. To establish such an experimental model, three factors need to be initially investigated: the ‘trans-generational’ effect of maternal exposure agents that have been linked with increased leukaemia risk in offspring, the role of the barrier between mother and foetus and the nature of DNA damage in the foetal blood.

In this study, DNA damage in cord blood and bone marrow is quantitatively and qualitatively described, using chromatin lesions or recruited DNA repair proteins, including phosphorylated histone H2AX (γ-H2AX) to mark double strand breaks, Fanconi Anemia Complementation Group D2 (FANCD2) to mark DNA cross-links, KU70 and KU80 which are involved in non-homologous end joining (NHEJ), Replication Protein 32 kDa subunit (RPA32) which is recruited during homologous recombination, X-ray Repair Cross-Complementing protein 1 (XRCC1) to mark single-strand breaks, and p53 Binding Protein 1 (53BP1) as a generic DNA damage sensor. Following the DNA damage, the cell must survive, without full recovery of its DNA damage by means of DNA repair, and have or obtain clonogenic potential, for it to establish a pre-leukaemic clone.

The interface between mother and foetus during pregnancy, the placenta, plays a key role in foetal programming^19,20^. Specifically, the barrier between maternal and foetal blood in the placenta is established by trophoblast cells. During the first trimester, a bilayer of trophoblast lines the chorionic villi of the placenta that bathe in a pool of maternal blood^21^. Foetal-maternal transport takes place across this trophoblast barrier. We have previously shown that some agents (nanoparticles, altered oxygen and pharmacological inhibition of the electron transport chain), which cause oxidative stress to the trophoblast barrier, will also cause it to release factors that induce DNA damage in cells on the opposite side of the exposed surface of the barrier^22–26^. In these previous studies, the chemicals that are associated with leukaemia (see Table 1) and the effects of radiation and mimics of infections were not studied.

We have also previously shown in mice that intravenous injection of a mitochondrial antioxidant, mitoquinol (MitoQ), attached to a drug delivery nanoparticle, allows the antioxidant to enter the placental barrier without reaching the foetus, and not only prevent oxidative stress in the placenta and placental secretions, as caused by maternal hypoxia, but also prevent altered foetal brain and cardiovascular development^26^. Considering that some of the suggested leukaemia-initiating agents also cause oxidative stress^27,28^, we hypothesised that such agents might cause the barrier to release factors that cause DNA damage and/or chromosome aberrations in cord blood cells, used here to model foetal haematopoietic cells. Current thinking might suggest that chemicals pass through the placental barrier to reach the foetus and so damage the blood by a direct exposure. We have examined here whether they might instead, or as well, damage the foetal blood indirectly, by initiating DNA damaging signalling between the placenta and the foetal blood. We have used *in vitro*, *ex vivo* and *in vivo* models of the placenta to simulate what might occur during establishment of DNA damage in the developing foetal haematopoietic system, and we have tested the efficacy of a nanoparticle-bound antioxidant in preventing DNA damage. We have shown that the placenta can release DNA damaging factors in response to chemical and radiation exposure, to which blood cells are selectively sensitive. This lesion could represent an initiating hit, in the sense that the DNA damage is enhanced after a ‘secondary hit’, in the form of an induced inflammatory response, using our *in vivo* model. Administration of MitoQ -bound nanoparticles to the mother during pregnancy, or to the placental barrier in culture, prevented this DNA damage.

## Results

### Differential DNA damage response between fibroblasts and cord blood exposed to trophoblast conditioned media *in vitro*

**Figure 1:**
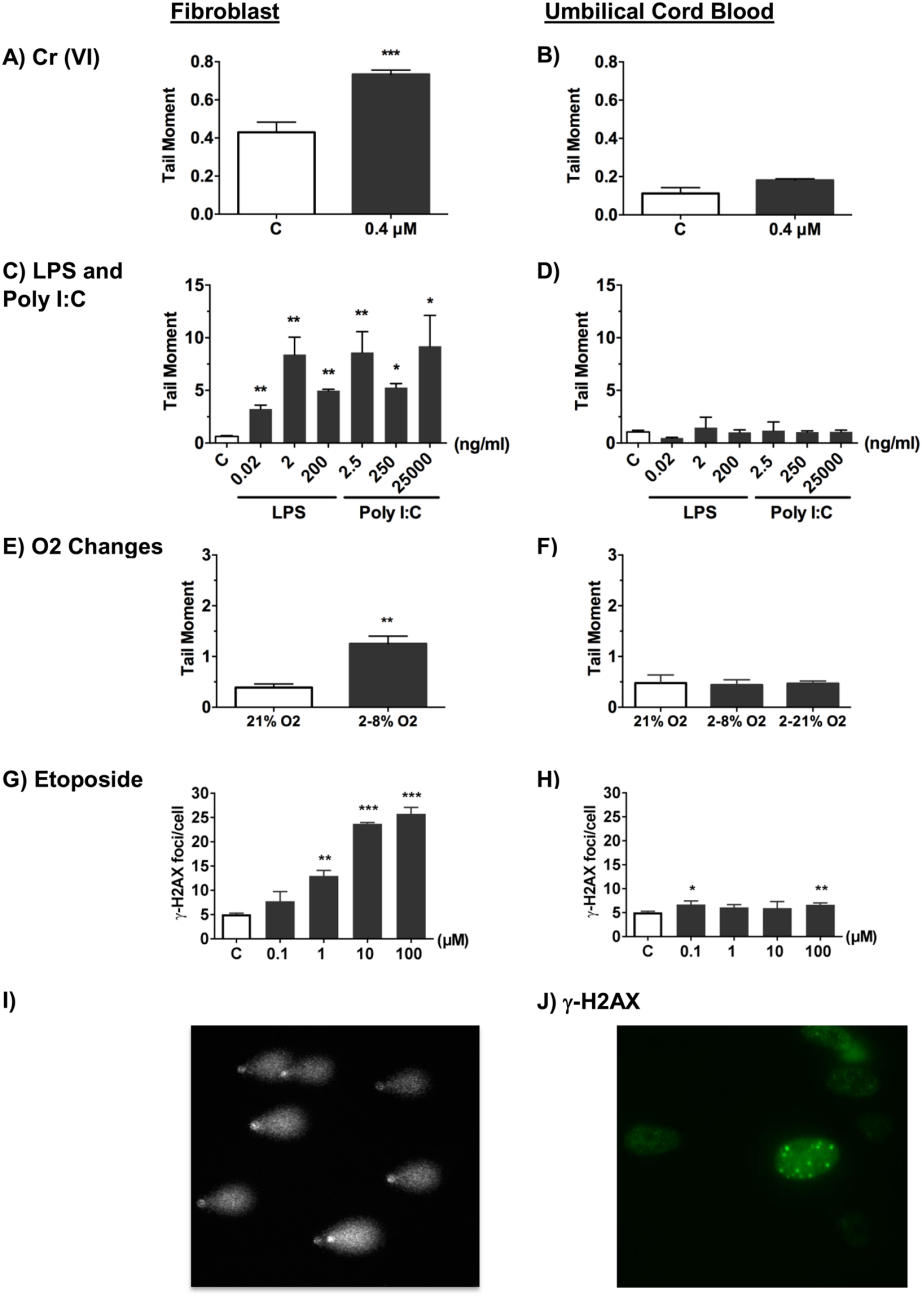
Differential DNA damage response between fibroblasts and cord blood after exposure to conditioned media from BeWo barriers. The level of DNA damage in fibroblasts as recorded by either the mean tail moment, using the alkaline comet assay (**A**–**F**), or immunocytochemical analysis of the mean number of γ-H2AX foci per cell (DNA double-strand breaks). (**G**,**H**) Values are shown for fibroblasts (left hand column) and umbilical cord blood (UCB) mononuclear cells (right hand column) after a 24 hour exposure to conditioned media below BeWo barriers resting on transwell inserts. The top surface of BeWo barriers was exposed to culture media without (open histograms) or with (shaded histograms) (**A**,**B**) 0.4 uM Cr(VI); (**C**,**D**) LPS or PolyI:C (ng/ml); (**E**,**F**) altered levels of oxygen; (**G**,**H**) etoposide (0.1, 1, 10 and 100 μM). Representative images of (**I**) comet assay and (**J**) γ-H2AX immunostaining are shown. Plots represent the mean ± SD of 3 independent experiments. n = 3. *p < 0.05, **p < 0.01, ***p < 0.001 as determined by unpaired two-tailed student’s *t*-test.

We first tested whether a simple *in vitro* model of the placental barrier would release a DNA damaging factor if it was exposed to agents that may cause leukaemia. A bilayered barrier of BeWo trophoblast cells resting on transwell inserts was used as the placental barrier model^25,29^. The top surface of the barrier was exposed for 24 hours to the putative leukaemic agents and the tissue culture media below the barrier (conditioned media, CM) was collected. Human fibroblasts were then exposed for 24 hours to the conditioned media, using the fibroblasts as a standard cell type^23,25^ with which to measure the amount of DNA damage induced by factors released into the conditioned media. We compared the damage caused by conditioned media in fibroblasts to the damage recorded in umbilical cord blood cells in an identical set up. The increase in DNA damage was recorded using the alkaline comet assay (Fig. 1I) to detect single and double strand breaks and alkaline labile sites, and γ-H2AX as a marker of DNA double strand breaks (Fig. 1J). The conditioned media below barriers exposed to Cr (VI) ions (Fig. 1A), lipopolysaccharide (LPS) (a potent immunostimulant found in the cell wall of Gram negative bacteria) and polyinosine-polycytidine (PolyI:C) (a synthetic double-stranded RNA that mimics viral infection) (Fig. 1C), and etoposide (a chemotherapeutic agent that acts by inhibiting DNA topoisomerase II) (Fig. 1G) all caused significant DNA damage in human fibroblasts. Previous research using the same concentration of Cr (VI) ions (0.4 μM) showed that only a small concentration of Cr (VI) ions passed through the bilayered BeWo barrier and that this was too low to cause DNA damage in fibroblasts^23^. This suggested that the damage was due to release of DNA-damaging agents from the barrier rather than a passage of Cr(VI) across the barrier and into the conditioned medium. To explore this possibility further, we exposed the barriers to hypoxia followed by reoxygenation, inducing a hypoxia response, validated by increased protein level of hypoxia- inducible factor 1-alpha (Fig. S1). Here, no chemical would be present to pass through a barrier. Nonetheless, the conditioned media caused DNA damage in fibroblasts (Fig. 1E). This points to a DNA damaging factor being released by the barrier, rather than an exposing agent passing through the barrier to damage the fibroblasts directly. We tested whether human cord blood mononuclear cells would also be damaged by these factors. Surprisingly, and unlike fibroblasts, there was no increased DNA damage in human cord blood cells that had been exposed to conditioned media below barriers whose top surface had been exposed to Cr (VI) (Fig. 1B), PolyI:C or LPS (Fig. 1D), altered oxygen levels (Fig. 1F) or etoposide at several doses (Fig. 1H).

This suggests that there is a DNA damaging factor which is released by the placental barrier *in vitro* in response to certain agents, to which fibroblasts are sensitive and cord blood cells are not.

### DNA damage in cord blood after indirect exposure to leukaemogenic agents *in vitro*

**Figure 2:**
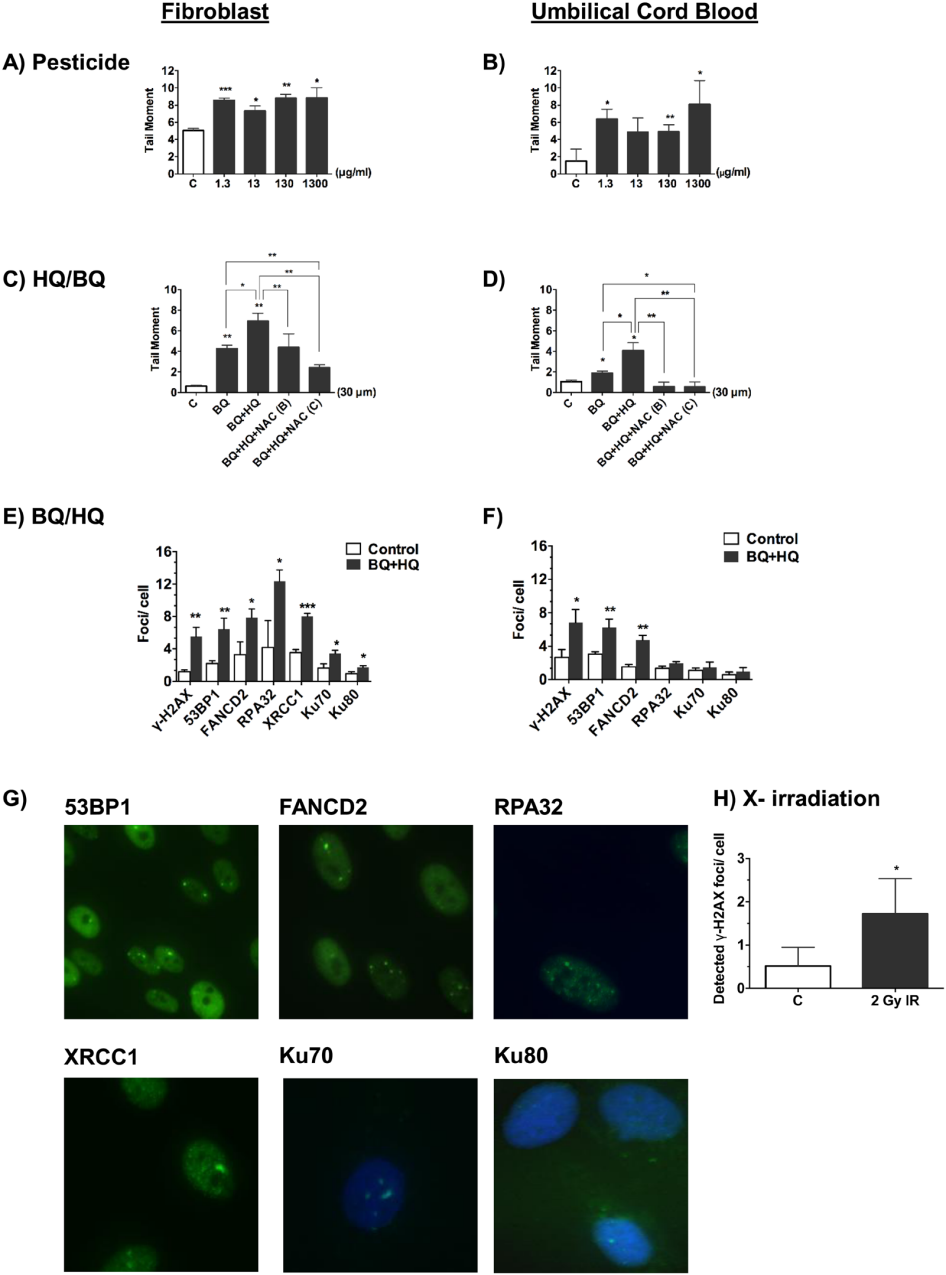
DNA damage in cord blood and fibroblasts after exposure to conditioned media from BeWo barriers treated with leukaemogenic agents. The level of DNA damage as recorded by either the mean tail moment, using the alkaline comet assay (**A**–**D**), or immunocytochemical analysis of the mean number of γ-H2AX foci per cell (DNA double-strand breaks) (**E**,**F**,**H**), together with the mean number of foci per cell of 53 BPI (DNA double-strand breaks), FANCD2 (DNA interstrand crosslinks), RPA32 (homologous recombination), XRCC1 (DNA single-strand breaks), Ku70 and Ku80 (non-homologous end joining) (**E**,**F**). Representative images of these markers are shown (**G**). Values are shown in for fibroblasts (left hand column) and umbilical cord blood (UCB) mononuclear cells (right hand column) after a 24 hour exposure to conditioned media below BeWo barriers resting on transwell inserts. The top surface of BeWo barriers was exposed to culture media without (open histograms) or with (shaded histograms) (**A**,**B**) pesticide comprising of pyrethroids with piperonyl butoxide (1.3 µg/ml, 13 µg/ml, 130 µg/ml and 1300 µg/ml); (**C**,**D**) benzene metabolites consisting of benzoquinone (BQ, 30 μM) with or without hydroquinone (HQ, 30 µM) with or without the antioxidant N-acetyl cysteine (NAC, 10 mM) which was either applied to the barrier (**‘B’**) or to the fibroblast or cord blood cells directly (**‘C’)**; (**E**,**F**) benzoquinone (BQ, 30 μM) and hydroquinone (HQ, 30 μM); (**H**) The level of DNA damage in UCB cells is shown after direct exposure to X-irradiation (2 Gy). Plots represent the mean ± SD of 3 independent experiments. n = 3. *p < 0.05, **p < 0.01, ***p < 0.001 as determined by unpaired two-tailed student’s *t*-test.

To assess if this differential response between cord blood and fibroblasts holds true for leukaemic agents, we exposed BeWo barriers to agents that have been linked more extensively and more convincingly to leukaemia initiation: pesticides, benzene and radiation (see Table 1). Similar to the experiments discussed in Fig. 1, DNA damage was measured in cord blood and fibroblasts after *in vitro* exposure to BeWo collected media. The conditioned media below barriers exposed to piperonyl butoxide and pyrethroid (a common pesticide formula) (Fig. 2A), and benzoquinone (BQ) and hydroquinone (HQ) (benzene metabolites) (Fig. 2C), caused significant DNA damage in fibroblasts. Using Selective Ion Monitoring (SIM) in our study, we found no evidence of hydroquinone or benzoquinone in the conditioned media below the barrier after exposure of these chemicals above the barrier (Fig. S2). In addition, the integrity of BeWo cell barriers was measured by trans-epithelial electrical resistance (TEER) (data not shown) and by testing the endothelial permeability to FITC-BSA (Fig. S3) and no evidence of a change in permeability or electrical resistance was observed. This is also in keeping with a DNA damaging factor that is released from BeWo cell barriers. However, unlike the previous exposures (Fig. 1), here there was a significant increase in DNA damage in cord blood cells, as well as fibroblasts, if they were exposed to condioned media below barriers whose top surface had been exposed to pesticides (Fig. 2B), and benzoquinone with or without hydroquinone (Fig. 2D). The cord blood cells also showed increased DNA damage if they were directly exposed to irradiation (Figs 2H and S4), in keeping with the literature that singles out radiation as a known cause for leukaemia^30–33^. Together, these findings suggest that human cord blood cells are selective in their response to the DNA damaging factors in the conditioned medium.

A comparison was made of the protective effect of the antioxidant N-acetyl cysteine (NAC) on the signalling of barriers exposed to benzoquinone and hydroquinone to both fibroblasts and cord blood. Applying N-acetyl cysteine either to barriers (B) (Fig. 2C,D) or directly to the cells (C) (Fig. 2C,D) that were exposed to the conditioned medium, partly reduced the DNA damage in fibroblasts (Fig. 2C) but completely abolished the damage in cord blood cells (Fig. 2D) suggesting that there might be a second (non-oxidative) molecule that was released from BeWo barriers that would damage fibroblasts but not cord blood cells. In addition, there was a different pattern of DNA damage in fibroblasts (Fig. 2E) compared to cord blood cells (Fig. 2F) after exposure to conditioned medium below hydroquinone and benzoquinone exposed barriers. Although there was a significant increase in γ-H2AX, 53BP1 and FANCD2 in both fibroblasts and cord blood cells, the levels of RPA32, Ku70 and Ku80 were only elevated in fibroblasts (see also Fig. 2G). This suggests that cord blood cells differ in their DNA damage response from fibroblasts, displaying a more selective reponse to exposures and a more restricted DNA damage pattern.

### Numerical and structural cytogenetic analysis after indirect exposure of cord blood mononuclear cells to benzene metabolites and pesticides

Having seen DNA strand breaks in cord blood cells, it was of interest to see whether this might be accompanied by an increase in cytogenetic abnormalities. The commonest karyotypic abnormalities in childhood leukaemia are charaterised by hyperdiploidy (35%) or ETV6/RUNX1 fusion (25%), which are mutually exclusive events^6^. Another 6% of cases is characterised by hypodiploidy^34^. The karyotypic abnormality is a strong predictor of the survival of the patient: hypodiploidy having a worse prognosis than hyperdiploidy or ETV6/RUNX1 fusion.

**Figure 3:**
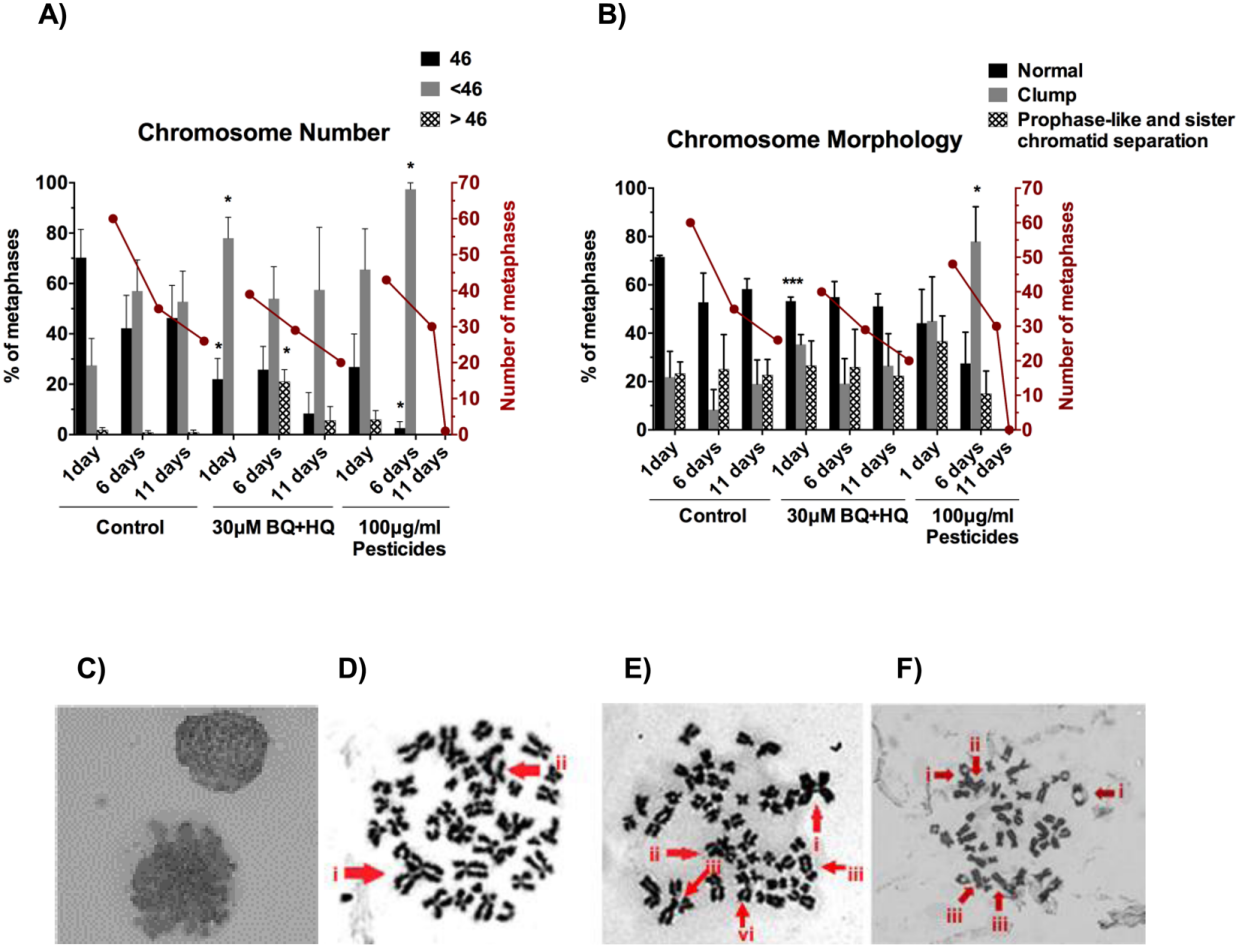
Cytogenetic analysis of mononuclear cord blood cells after exposure to conditioned media below BeWo barriers exposed to benzene metabolites and pesticides. Numerical (**A**) and structural (**B**,**C**) analyses of metaphase spreads from cord blood cells at 1, 6 and 11 days after a 24 hour exposure to the conditioned media below BeWo barriers, whose top surface had been exposed to plain cell culture media (control), either 30 μM hydroquinone and 30 μM benzoquinone (30 μM BQ + HQ) or 100 μg/ml pyrethroids with piperonyl butoxide for 24 hours (100 μg/ml pesticides). (**A**) Percentage of metaphase spreads with 46 (normal), less than 46 (hypodiploid) or more than 46 (hyperdiploid) chromosomes (histograms) and number of metaphases per slide (lines). (**B**) Percentage of metaphases in which chromosomes were well spread (normal) or clumped together, and number of metaphases per slide (lines). Histograms represent mean and SD (n = 4). (**C**) Representative metaphase spreads displaying chromosome clumping. (**D**–**F**) Representative abnormal chromosomal events, taken one day after exposure to conditioned media (CM) below benzene or pesticide exposed BeWo barriers: (**D**) Metaphase containing numerous structural chromosome abnormalities, the most striking of which are: (i) Tri-radial chromosome as a result of chromatid interchange; (ii) Fusion between two chromosomes; (BQ + HQ) (**E**) Metaphase spread with (i) Quadri-radial chromosome; (ii) asymmetrical tri-radial chromosome; (iii) two examples of double chromatid breakage and (vi) Satellite associated chromosome (pesticide) (**F**) Metaphase spread with (i) two examples of satellite association chromosomes; (ii) Chromatid exchange between two different chromosomes; (iii) Two examples of di-centric chromosomes (pesticide). Plots represent the mean ± SD. n = 3. *p < 0.05, ***p < 0.001 as determined by unpaired two-tailed student’s *t*-test.

Indirect exposure to benzoquinone and hydroquinone across the BeWo barrier led to a significant increase in numerical chromosome aberrations in metaphases in the short- (1 day) and medium-term (6 days) after a single 24 hour exposure to the conditioned medium from below the barrier (Fig. 3A). There was a significant increase of hypodiploidy one day after exposure and a significant increase of hyperdiploidy 6 days after exposure. The mitotic rate (measured as the number of metaphases per slide) remained similar to control levels (Fig. 3A). Indirect exposure to pesticides across the barrier also caused a significant increase of chromosome aberrations, specifically hypodiploidy measured after 6 days. There was a greater frequency of metaphases in which the chromosomes were clumped together with nuclear membranes being faintly present 6 days after indirect exposure to pesticide (Fig. 3B,C). This could indicate failure of nuclear envelope breakdown and will hinder cell cycle progression, which could have caused the subsequent drop in mitotic rate. Further examples of abnormal chromosome morphology after indirect exposure included triradial, quadriradial, and dicentric chromosomes, double chromatid breakage, satellite-associated chromosomes, and chromatid exchange between two different chromosomes after exposure to the conditioned medium (Fig. 3D,F). To check for genetic lesions that are not involved in leukaemia initiation, telomeric fusion events were analysed. There was no evidence of end-to-end joining in the form of telomere fusions, when measured using single telomere length analysis (STELA) (Fig. S5).

Despite the levels of DNA damage and cytogenetic abnormalities, the cord blood cells exposed to CM from benzene or pesticides did not show altered colony forming potential (Fig. S6) or major changes in lineage output (Fig. S7). This suggests that the majority of stem/progenitor cells retained their functionality.

### Collected media from placentae exposed *in vivo* can send a DNA damage signal to neonatal bone marrow cultured *ex vivo*

**Figure 4:**
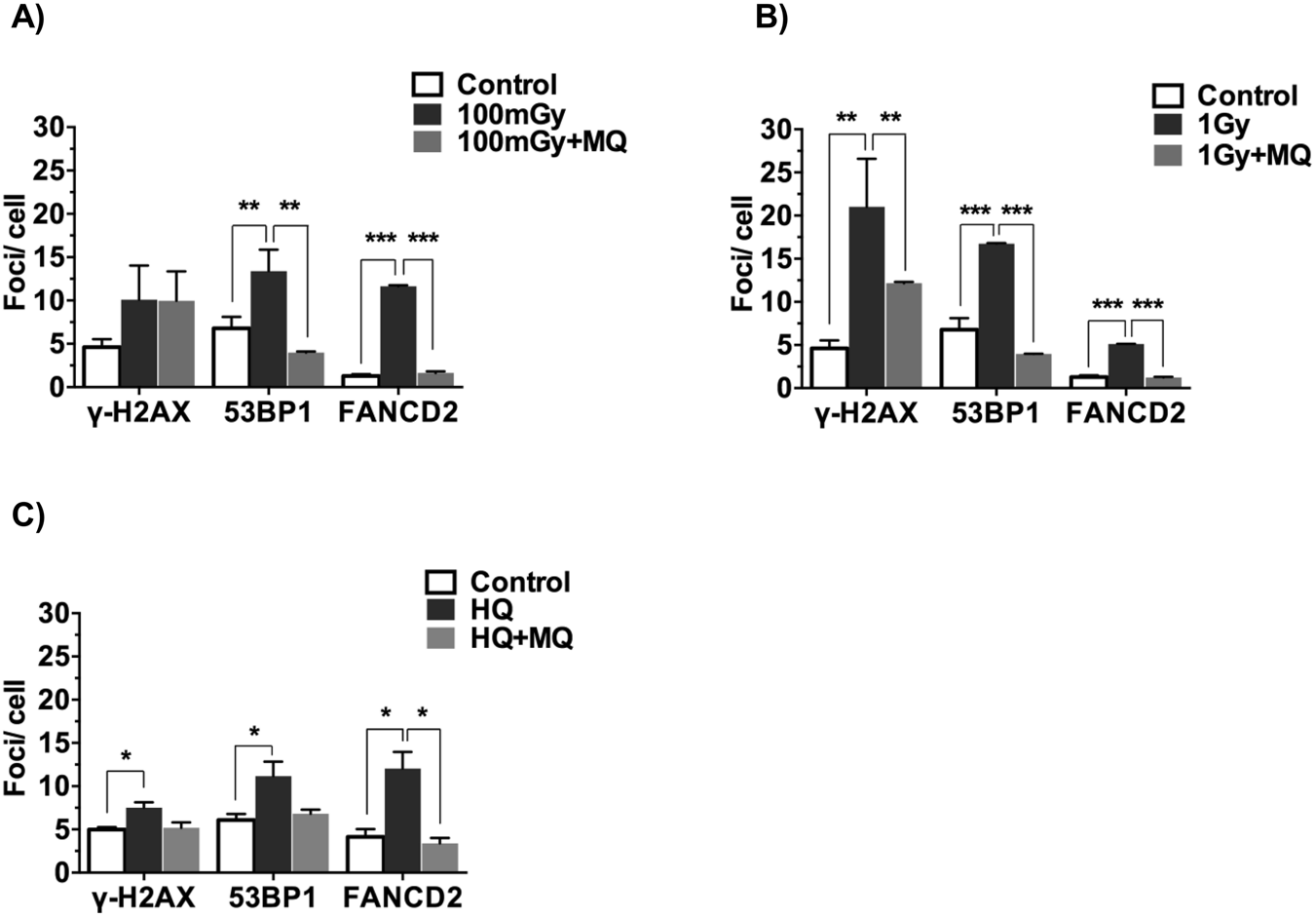
*Ex vivo* model of placental signalling and its contribution to DNA damage signal propagation using γ-H2AX, 53BP1 and FANCD2 as DNA damage markers. (**A**,**B**) The placentae were removed from mice that were exposed to 0.1 or 1 Gy X-irradiation at GD12 or to (**C**) hydroquinone (at GD12) (50 mg/kg body weight in H_2_O) by oral gavage. Bone marrow cells from age-matched female C57Bl/6J were exposed for 24 hours *in vitro* to conditioned media. This conditioned media was generated by bathing the placentae for 24 hours in fresh culture medium, with or without addition of MitoQ-bound nanoparticles (MQ, 0.5 μM). The bone marrow cells were then screened for the level of DNA damage. Four placentae were used per mouse. Four mice per condition were investigated. n = 4. *p < 0.05, **p < 0.01 and ***p < 0.001, compared to untreated controls (unpaired two-tailed student’s *t*-test).

We next tested whether the placenta might be able to send a DNA damaging signal if the mother was exposed *in vivo* to an agent known to be associated with increased risk of leukaemia. Pregnant mice were exposed at gestational day (GD) 12 to either 0.1 or 1 Gy of whole body X-irradiation, sacrificed 4 hours later and the placentae were removed. Each placenta was placed into tissue culture media with or without an antioxidant treatment for 24 hours to condition the culture media. Because some of the experiments that follow were done *in vivo*, we used the antioxidant MitoQ bound to nanoparticles as a convenient way of treating the placenta and not the foetus^35^, which has not yet been achieved with N-acetyl cysteine. A 24 hour *ex vivo* exposure of mouse bone marrow cells (from 4 week old mice) to the conditioned media caused an increase in DNA damage, including double strand breaks (γ-H2AX and 53BP1 foci) and DNA cross-links (FANCD2) (Fig. 4A,B). This confirmed that DNA damaging factors are released by a placenta exposed to radiation *in vivo*, to which haematopoietic cells are responsive. In a subsequent experiment, pregnant mice were exposed to HQ (only, as BQ is metabolised from HQ *in vivo*) at GD12 for 24 hours through oral gavage, with or without an intravenous injection of the MitoQ nanoparticles. Again, the placentae were removed (six days later, at GD18) and placed in tissue culture media for 24 hours to condition the culture media. This conditioned media caused an increase in markers of DNA damage and cross-linkage in mouse bone marrow cells after a 24 hour exposure (Fig. 4C). In both experiments, after maternal exposure to radiation or to HQ, the MitoQ nanoparticles reduced the level of DNA damage (particularly 53BP1) and cross-linkage in the mouse bone marrow cells. No HQ was detected in the conditioned media after *in vivo* injection. In view of this, and because irradiation would not have been retained in the placenta to leak into the tissue culture conditioned media, we can conclude that the placenta can send a DNA damaging signal if it is exposed *in vivo* to an agent that is associated with a risk of leukaemia and that this can be prevented by means of antioxidant treatment.

### Maternal hydroquinone exposure causes damage to foetal bone marrow *in vivo* that is reinstated after immunostimulation during the equivalent of early childhood

**Figure 5:**
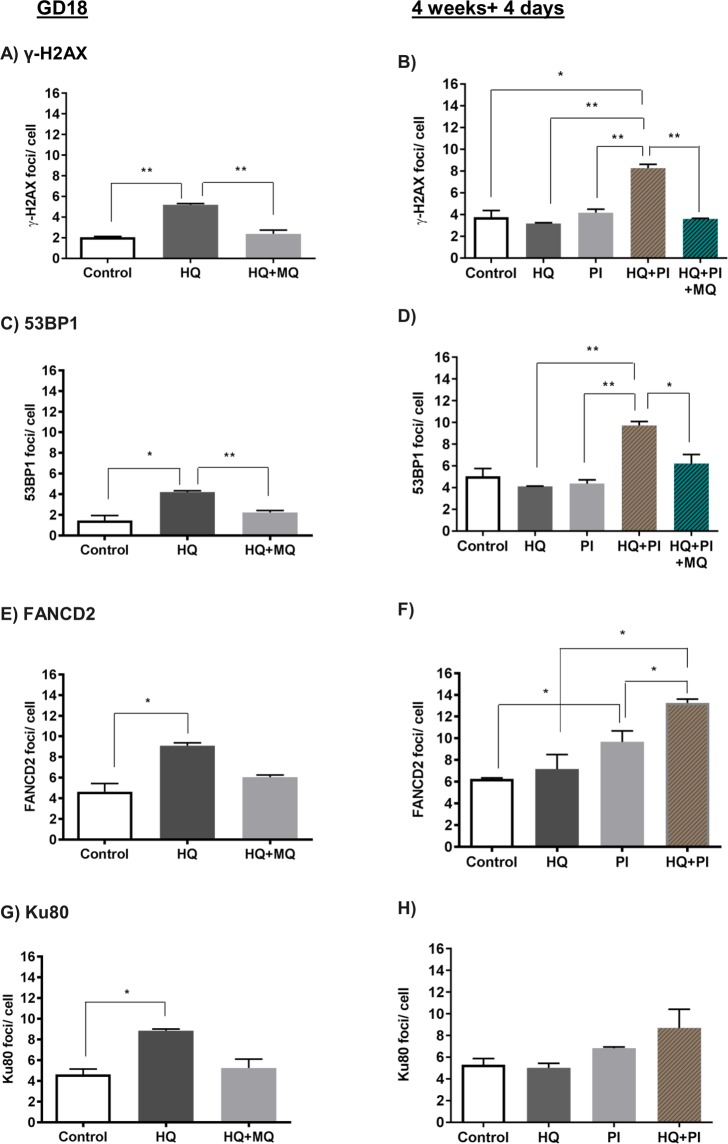
The effects of maternal exposure to hydroquinone ± MitoQ-bound nanoparticles, with or without postnatal exposure to PolyI:C, on DNA damage in the bone marrow of the offspring. Pregnant mice were exposed to vehicle (H_2_O) (control, open histograms), hydroquinone (HQ) or hydroquinone and MitoQ nanoparticles (HQ + MQ) (shaded histograms) at GD12 and the level of DNA damage was measured in bone marrow cells from the offspring at delivery (GD18, left hand column) or at 4 weeks + 4 days postnatally (right hand column), with (+PI) or without an exposure of the offspring to PolyI:C at 4 weeks postnatally. Graphs show the mean ± SEM number of foci per cell of γ-H2AX (**A**,**B**), 53BPI (**C**,**D**), FANCD2 (**E**,**F**) and Ku80 (**G**,**H**). Four placentae were used per mouse and 4 mice were investigated per condition. n = 4 *p < 0.05, **p < 0.01, ***p < 0.001 as determined by unpaired two-tailed student’s *t*-test.

Subsequently we examined whether the mononuclear cells in the bone marrow of the foetus (GD18) would be damaged *in vivo*, if the mother had been exposed to HQ through oral gavage at GD12, with or without a concurrent intravenous injection of MitoQ nanoparticles. In our previous experiments we showed that these MitoQ nanoparticles would prevent increased oxidative stress in the placenta but not reach the foetus^35^. The results showed that, like the *ex vivo* experiments (Fig. 4), there were significant increases in markers of DNA damage as indicated by γ-H2AX, 53BP1 (Fig. 5A,C), FANCD2 (Fig. 5E) and Ku80 foci (Fig. 5G) and that this could be prevented or was no longer apparent after the intravenous injection of nanoparticle-bound antioxidant.

Having established that the foetal bone marrow could be damaged *in vivo* by a single maternal exposure to the benzene metabolite and prevented by maternal injection of antioxidant, we next explored whether this damage had the potential to act as a first hit in that it might be altered or augmented by a second hit occurring postnatally. In order to explore this, we repeated the *in vivo* experiments but this time kept the offspring from the exposed mother until 4 weeks postnatally. The offspring were then given an intraperitoneal injection of PolyI:C to mimic a second hit in the form of a viral infection in early childhood and examined 4 days later. In these experiments, the bone marrow of the offspring without PolyI:C treatment (labelled as HQ) no longer showed overall increase in DNA damage (Fig. 5B,D), cross-linkage (Fig. 5F) or non-homologous end joining (Fig. 5H), suggesting that the damage had been mostly repaired. Strikingly though, if the offspring were exposed to the viral mimic (HQ + PI), there were increased double strand breaks (Fig. 5B,D) and cross-links (Fig. 5F) in the bone marrow cells. The increase of double strand breaks was prevented by the maternal exposure to antioxidant nanoparticles during pregnancy and was not seen after PolyI:C treatment at 4 weeks without prior exposure to HQ during pregnancy (PI). We therefore conclude that this intrauterine exposure during pregnancy, whose effect can be prevented by the maternal exposure to nanoparticle-bound antioxidant, has a feature of a first hit in that it can be revitalized or augmented or made effective by a relevant second hit during the equivalent of early childhood.

**Figure 6:**
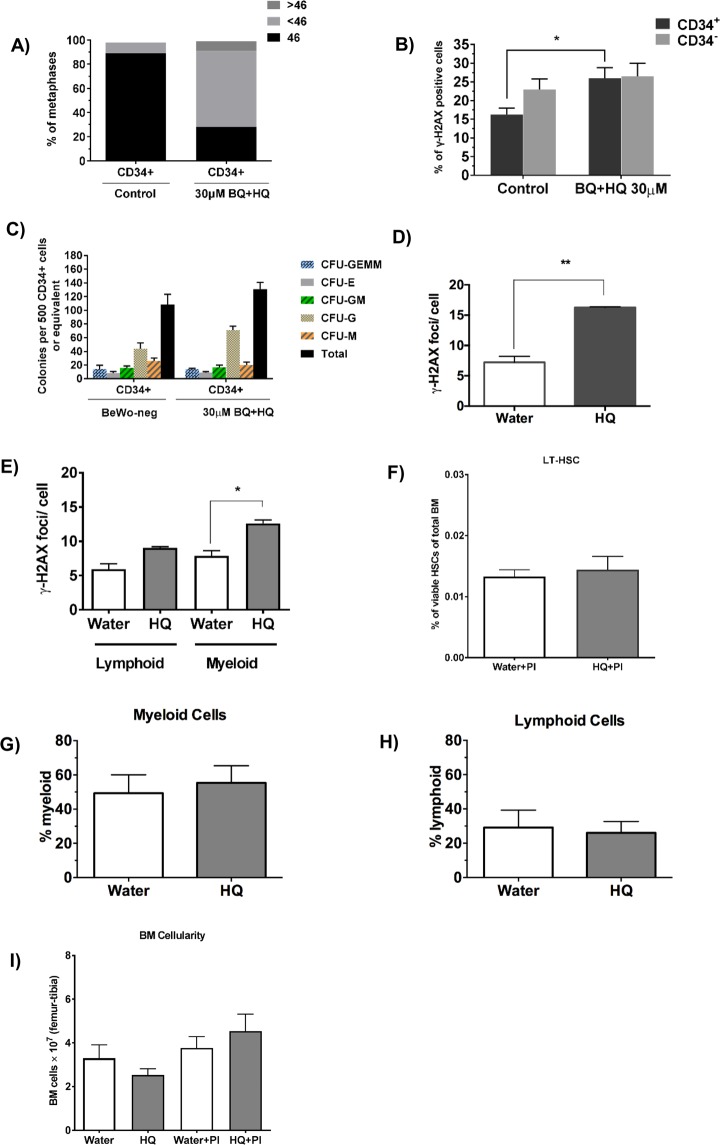
Assessment of DNA damage in the stem and progenitor cell compartment. CD34^+^ cord blood cells were FACS sorted after a 24 hour exposure to plain BeWo CM (control) or from BQ + HQ exposure (30uM BQ+HQ). Metaphase spreads indicate numerical chromosome abnormalities in CD34+ cells three days after exposure (**A**). Flow cytometric analysis of γ-H2AX staining within proliferating (ki-67^+^) CD34^+^ and CD34^−^ fractions (**B**). Colony forming unit assay of CD34^+^ cells plated one day after exposure (**C**). Pregnant females were injected with either vehicle (H_2_O) or hydroquinone at GD12. Mean γ-H2AX foci in HSCs that were taken from pups and induced to proliferate with IL-1 without PolyI:C treatment (**D**). The pups were injected with PolyI:C 4 weeks after birth and their BM harvested 4 days later for examination of the mean number of γ-H2AX foci in lymphoid and myeloid cells (**E**), HSC frequency within the BM (Lin^−^/Sca1^+^/ckit^+^/Flk2^−^/CD48^−^/CD150^+^) (**F**), myeloid cells (Gr1^+^ and CD11b^+^) (**G**) and lymphoid cells (CD3^+^ and B220^+^) (**H**) and bone marrow cellularity (**I**). Plots represent the mean ± SE. Four mice were investigated per condition. n = 3. *p < 0.05, **p < 0.01 as determined by unpaired two-tailed student’s *t*-test.

This result is unlikely to be a consequence of mutagenesis induced by VDJ rearrangements upon immune stimulation because there was no increase in double strand breaks in the lymphoid fraction of the bone marrow specifically (Fig. 6E). One possible explanation is that, although most of the intrauterine DNA damage would have been repaired or removed by 4 weeks postnatally, a small population of haematopoietic stem/progenitor cells has retained damage and these cells gained proliferative advantage upon activation with PolyI:C, passing on their DNA damage to progeny.

### Damage to the stem/progenitor cell compartment

To test the hypothesis that DNA damage caused by benzene-induced placental signalling is retained in stem/progenitor cells, we further characterised the damage in CD34^+^ cord blood cells after *in vitro* exposure and in various lineages of the mouse haematopoietic system after *in vivo* exposure. It has been shown that human leukaemia-initiating cells are often found within the CD34^+^ fraction^36–38^. For this reason, CD34^+^ cells were enriched by fluorescence activated cell sorting (FACS) after a 24 hour exposure to BeWo CM from BQ + HQ exposure. Metaphase spreads of CD34^+^ cells displayed a remarkable increase in predominantly hypodiploid, but also hyperdiploid, metaphases after exposure to the collected media compared to controls, at the expense of euploid cells (Fig. 6A). Furthermore, it was shown that proliferating (Ki-67^+^) CD34^+^ cells are especially sensitive to damage induced by BeWo CM after benzene exposure, shown by a significant increase in γ-H2AX positive cells in the proliferating CD34^+^ but not CD34^−^ compartment (Fig. 6B). Despite CD34^+^ cells harbouring such damage, their colony forming potential was not affected, suggesting that multi-lineage clonogenic potential is retained (Fig. 6C).

To test whether HQ treatment *in vivo* had resulted in the accumulation of DNA damage in the HSC pool of the offspring, which is then expanded during stress-induced HSC proliferation, with the DNA damage being passed on to the progeny, we employed an *in vitro* assay for inflammatory stress-induced HSC proliferation, using interleukin-1 (IL-1) as a pro-inflammatory signal^39^. This assay involves culturing purified mouse HSCs (Lin^−^/Sca1^+^/ckit^+^/Flk2^−^/CD48^−^/CD150^+^) in the presence of IL-1, which induces HSC proliferation and differentiaton towards the myeloid lineage. Pregnant females were again treated with either H_2_O (vehicle) or HQ at GD12 and the bone marrow of the pups was harvested at 4 weeks of age. HSCs were then isolated by FACS and grown in culture for 8 days in the presence of IL-1. At the end of the culture, the cells were spotted on slides and stained for γ-H2AX foci (Fig. 6D). The results revealed a highly significant increase of DNA damage in the cells from HQ-treated mice, demonstrating that HSCs from the offspring of HQ-treated mothers had indeed retained DNA damage in their HSC population which is maintained upon proliferation and differentiation following an inflammatory response. The bone marrow of pups harvested at 4 weeks postnatally and after polyI:C treatment also showed increase in DNA damage in the myeloid compartment if mothers were exposed to HQ (Fig. 6E). It cannot be deduced from the *in vivo* data, however, if this DNA damage occured in mature myeloid cells or whether the damage is inherited from stem/progenitor cells through proliferation and differentiation.

### Cellular distributions

FACS analyses revealed that there was no significant increase in bone marrow cellularity (Fig. 6I) and in the size of the myeloid (Gr1^+^ and CD11b^+^) and lymphoid (CD3^+^ and B220^+^) compartments (Fig. 6G,H, respectively) in the bone marrow of the 4 week old offspring, after maternal HQ exposure *in vivo*. This is consistent with the observation that the size of the HSC pool in the offspring was not affected by maternal HQ treatment (Fig. 6F).

The bone marrows were also examined with histology and cytology. All bone marrows in paraffin sections, both experimental and control, were markedly hypercellular at this age, with predominat hyperplasia in the myeloid and megakaryocyte lineages (Fig. S8A–D). Only minor morphological differences were seen *in vivo* between experimental and control samples; the megakaryocytes were more hyperlobulated in experimental marrows than in control. After hydroquinone treatment, without PolyI:C, there were fewer erythroid islands and scattered myeloblasts compared to the other marrows. No small B cells (CD20^+^) were seen although a small number were present in control and other experimental marrows. After PolyI:C treatment, there were scattered myeloblasts and an increase of T cells (CD3^+^) from 1% to 5%. The bone marrow aspirates were also hyperplastic with a left shifted myeloid lineage (Fig. S8E,F). This was more noticeable in experimental aspirates compared to control. In conclusion, the *in vivo* exposed bone marrow cells appear to behave in a similar manner to the *in vitro* exposed cord blood. Despite the increase in DNA damage, there is little evidence that the exposures caused a profound shift in proliferation or differentiation capacity. This is in line with our hypothesis because presence of pre-leukaemic mutations alone is not sufficient to alter stem/progenitor cell behaviour. This is evident by the large proportion of healthy people harbouring ETV6/RUNX1 fusion genes in their haematopoietic cells, without ever developing haematological malignancy. Only 1% of people carrying initiating mutations develop overt leukaemia, after encountering a secondary hit during a window of susceptibility^40^.

## Discussion

In this paper, we have set out to examine whether there is any evidence that *in utero* damage to haematopoietic cells could be caused by the placenta releasing factors in response to suspected leukaemia-initiating maternal exposures. We have noted previously that trophoblast barriers can release factors that cause DNA damage in human embryonic stem cells^22,41^ and human fibroblasts^23,25^ when the barriers have been exposed to agents that cause oxidative stress. We have also noted that this can be prevented by applying a soluble antioxidant (MitoQ) to the barrier or an agent that impairs cell-to-cell signalling within the bilayered trophoblast barrier^23,25^. In more recent experiments, we have also noted that an application of soluble MitoQ *in vitro* or an intravenous injection of MitoQ bound to nanoparticles *in vivo* (that enter into trophoblasts but do not reach the foetus)^35^ can prevent the placenta from releasing various factors, including those that can damage brain cells and prevent postnatal damage to the heart and brain (56, 57, 60). We therefore reasoned that a similar type of damaging communication might occur between the trophoblast and the foetal blood within the placental villi.

We have found it necessary to use three models, an *in vitro* model, an *ex vivo* model and an *in vivo* model as each model has its own advantages and disadvantages^42^. The *in vitro* model of BeWo cells allows a bilayered barrier of trophoblasts to be exposed in a polarized manner in keeping with *in vivo* exposure. Studies have reported considerable likeliness between BeWo cells and primary human trophoblast, with regards to architecture, secretions and receptor expression^43–45^. It is, however, comprised of a choriocarcinoma cell line. The *ex vivo* model allows the placenta to be exposed *in vivo* and has the correct architecture and cellular composition of the *in vivo* placenta but it is not possible to be certain here from which surface or cell type the DNA damaging signals might be sent. The *in vivo* model allows *in vivo* exposures to be made in a polarized fashion but here it is difficult to be certain that some exposures to the placenta, especially radiation, would not also expose the foetus. We have therefore employed all three models and drawn conclusions from each. Our *in vitro* model allows us to conclude that bilayered trophoblast barriers respond in the same way to the agents that are associated with increased risk of leukaemia as they did to previously studied insults^22,23,25,26,35^. After exposures on the top surface of the barrier, factors are released from the bottom surface of the barrier, which cause DNA strand breaks and chromosome aberrations in haematopoietic cells including stem/progenitor cells. As in the previous experiments^23,25^, the damage was prevented by treatment of the barrier with an antioxidant. It is interesting with regard to our hypothesis that the cord blood cells were selective in their response, compared to fibroblasts, and were only damaged by agents that have been strongly linked epidemiologically to an increased risk of leukaemia (see introduction).

Because the barrier *in vitro* and *ex vivo* released factors that caused DNA damage after exposure to a non-chemical agent, i.e. altered oxygen and X-irradiation, we can be confident that in these cases the barrier is releasing the DNA damaging agent and that there is no possibility of an exposing chemical passing through the barrier to cause direct damage to the cells on the other side. In the case of exposure to benzene metabolites, we found no evidence of the exposing chemical in the conditioned media and there was no change in barrier integrity (Figs S2, S3). This strengthens our original hypothesis^23^ that the placental trophoblast barrier can send a bystander-like signal to cause DNA damage in a neighbouring cell in a similar fashion to the radiation-induced bystander effect, where the receipt and emission of the signal is dependant on mitochondrial oxidative stress and where the effect can be noted *in vitro* and *in vivo*^46–49^. The prevention of this damage by the mitochondrial targeted antioxidant, which also prevents damage to heart and brain^35,50^, also strengthens this idea. The nature of the radiation-induced bystander signal is not known and several molecules have been implied^47^. The evidence in this paper also suggests that several molecules might be released from the barriers and that cord blood cells might be potentially sensitive to only some/one of them, which could explain their selective response. According to our hypothesis of bystander-like barrier signalling, it could also provide an explanation for why leukaemia is only caused by certain epidemiological agents.

As in our previous experiments, the characteristics of the *in vitro* or *ex vivo* barrier signalling were paralleled by the changes that were noted in an *in vivo* model. The *in vitro* experiments resulted in DNA double strand breaks, DNA cross-links and aneuploidy in the cells that were exposed to the conditioned media. As discussed, aneuploidy is the most prevalent karyotypic abnormality in cALL, followed by ETV6/RUNX1 fusion and other specific fusion events. Although these initiating mutations give rise to leukaemias with different clinical prognoses, they act similarly to one another in the sense that a single prenatal event is sufficient to render a cell pre-leukaemic, with leukaemia occuring in selected cases upon encounter of a secondary hit. Furthermore, there was evidence of damage to stem/progenitor cells without altering the differentiation capacity and whilst maintaining clonogenic potential. A similar behaviour is expected from pre-leukaemic cells that display grossly normal behaviour until a second hit is encountered. This process appeared to be prevented by antioxidant treatment using N-acetyl cysteine. The *in vivo* experiments also showed DNA double strand breaks and increased DNA cross-links in foetal bone marrow, with no marked change in the cellular compartments in the bone marrow, and again with evidence that this process might be prevented by treatment with an antioxidant.

We demonstrate some characteristics of a carcinogenic-like process with DNA damage, chromosome aberrations and damage to the stem/progenitor cell compartment. It would be of interest to determine in future experiments whether dysplastic or malignant changes would be observed in the longer term following injection with PolyI:C. What we have seen is a potential two-hit type of mechanism in which much of the DNA damage that occurred after a single *in utero* exposure was seemingly repaired by 4 weeks postnatally but where there was still residual damage, most likely concentrated in cells with clonogenic capacity, that was able to result in significant DNA damage when a viral immune stimulus or a stress stimulus, which causes proliferation of stem/progenitor cells, was encountered. Our observation of ‘trans-generational’ DNA damage therefore does mirror our current understanding of the two-hit hypothesis for childhood leukaemia.

Although the overall cure rate for paediatric acute leukaemia is high^51,52^, treatment can cause severe and long-term side effects, and certain subtypes remain troubled by poor prognosis^53,54^. Greater understanding of the disease aetiology is essential for paving the way to novel treatments or earlier intervention. The model described here can act as a useful tool in this endeavour and could be relevant to study other common leukaemia-initiating events, such as ETV6/RUNX1 and MLL fusion events. Should the type of process that we describe here prove to be of clinical relevance, there is a possibility that this damaging sequence might be potentially preventable through a strategic treatment with an antioxidant.

## Methods

### Chemicals

Chemicals were used at the following concentrations unless otherwise stated: HQ, 30 µM (Sigma-Aldrich, UK), BQ, 30 µM (Sigma-Aldrich), N-Acetyl-L-cysteine, 10 mM (Sigma-Aldrich), MitoQ, 0.5 mM (gift from M. Murphy, Antipodean Pharmaceuticals Inc.), Hexavalent Chromium ions, 0.4 µM (eCrater, UK), Polyinosinic–polycytidylic acid (Poly I:C), 2.5 ng/ml to 25 µg/ml (Sigma-Aldrich), Lipopolysaccharides (LPS), 0.02 ng/ml to 200 ng/ml (Sigma-Aldrich), epicatechin, 2 ng/ml to 20 ug/ml, quercetin, 100 nM to 100 µM (Sigma-Aldrich), etoposide, 100 nM to 100 µM (Sigma-Aldrich), pyrethroid standard mixture and piperonyl butoxide (pesticides), 1.3 µg/ml, 13 µg/ml, 130 µg/ml and 1300 µg/ml (Sigma-Aldrich).

Hypoxic exposures involved growing BeWo barriers in a modular incubator chamber (Billups-Rothenberg), flushed daily with 1% O_2_, 5% CO_2_ and N_2_ (balance), 37 °C for at least 4 min at a flow rate of 20 l min^−1^ and then sealed.

### Nanoparticles

An amphiphilic copolymer of poly (γ-glutamic acid) and L-phenylalanine ethylester (γ-PGAPhe) was synthesized as previously described^55^ using a 50% Phe grafting degree. 10 mg/ml of γ-PGA-Phe was dissolved in DMSO, added to equivalent volume of 0.15 M NaCl and dialyzed against distilled water. The dialyzed solutions were freeze-dried and resuspended in PBS (10 mg/ml). NPs were measured by dynamic light scattering (Zetasizer Nano ZS, Malvern Instruments, UK) as 180 nm diameter, polydispersity index 0.12, Zeta potential −20 mV.

γ-PGA-Phe NPs (10 mg/ml) were mixed with MitoQ (2 mg/ml) at equivalent volume in 0.2 M NaCl and incubated at 4 °C for 12 h. NPs were isolated by centrifugation, washed with PBS, and resuspended in PBS to 10 mg/ml.

### Cell culture

BeWo b30 cells were obtained from the University of Bristol (Dr Margaret Saunders) and cultured in Dulbecco’s modified eagle’s medium (DMEM) nutrient mixture F-12 Ham with phenol red, supplemented with 1% L-glutamine-penicillin- streptomycin (PSLG), 1% amphotericin B solution (AmpB) and 10% foetal bovine serum (FBS, Sigma-Aldrich) at 37 °C in 5% CO_2_. The cells were seeded at 1.12 × 10^5^ cells on polyester 0.4-mm-pore membranes in a transwell plate and grown as a barrier with regular medium change for 7 days.

Human umbilical cord units were obtained from NHS Cord Blood Bank, NHS Blood and Transplant (Filton, UK) with North Bristol research ethics committee approval and with informed patient consent. All methods were performed in accordance with the relevant guidelines and regulations. Mononuclear cells were obtained by Ficoll gradient centrifugation and red blood cells (RBC) were depleted using RBC lysis solution (Miltenyi Biotec, Bisley UK). The cells were maintained in Iscove’s Modified Dulbecco’s Medium (IMDM) supplemented with 10% FBS, 1% penicillin- streptomycin (Sigma) and 1% Glutamax at 5% O_2_, 5% CO_2_, 37 °C. For CD34 expansion culture, cytokines IL-3, G-CSF, TPO, IL-6, Flt3, SCF (in StemSpan SFEM media (STEMCELL Technologies, Cambridge, UK)) were added to the culture to maintain proliferation.

Primary BJ human fibroblasts were obtained from the American Type Culture Collection (ATCC) and maintained in minimal essential medium (Sigma-Aldrich) supplemented with 10% FBS (Invitrogen), 2% HEPES buffer (Sigma-Aldrich), 1% sodium pyruvate solution (Sigma-Aldrich), 1% penicillin- streptomycin solution and 1% L-glutamine (Sigma-Aldrich) at 5% O_2_, 5% CO_2_, 37 °C.

For the *ex vivo* stress-induced proliferation assay, 500 bone marrow HSCs (Lin^−^/Sca1^+^/ckit^+^/Flk2^−^/CD48^−^/CD150^+^), sorted independently from 3 pups whose mother had received vehicle (H_2_O) and from 3 pups whose mother had received HQ, were plated in StemPro34 (Invitrogen) containing Pen/Strep (50 U/ml), L-glutamine (2 mM), SCF (25 ng/ml), Flt3L (25 ng/ml), IL11 (25 ng/ml), IL3 (10 ng/ml), GM-CSF (10 ng/ml), Epo (4 IU/ml), Tpo (25 ng/ml) and IL1b (25 ng/ml). They were incubated at 37° in 5%CO_2_ for 8 days and 30% of the medium refreshed every other day.

### Measurement of DNA damage

DNA damage was measured using either quantitative immunocytochemistry (Q-ICH), the alkaline comet assay or Image stream.

### Quantitative immunocytochemistry

A panel of markers of DNA damage and repair was used which was comprised of antibodies against γ-H2AX; 53BP1 (Life Technologies, UK) (DNA double-strand break (DSB), FANCD2 (interstrand crosslinks (ICL), Ku70/Ku80 (non-homologous end joining (NHEJ)), RPA32 (gift from Dr. Abderrahmane Kaidi) (Homologous recombination (HR)) and XRCC1 (Single strand break (SSB)). All primary antibodies were purchased from Source Bioscience, USA unless otherwise stated. The fibroblasts were fixed over 10 min at room temperature (RT) in 2% paraformaldehyde (Sigma-Aldrich), washed in PBS and permeabilized over 10 min in 0.2% Triton (Sigma-Aldrich), washed twice in PBS, and blocked over 1 hr using 150 ml of PBS and 10% Bovine serum albumin (BSA) (Life Technologies,). Cells were stained overnight at 4 °C in 100 µl of primary antibody solution in PBS and BSA with the following concentrations: γ-H2AX (1:1000); 53BP1 (1:500), FANCD2 (1:500), Ku70/Ku80 (1:250), RPA32 (1:500) and XRCC1 (1:250). After cells were washed twice in PBS, 60 µl of secondary antibody solution (goat anti-rabbit IgG or donkey anti*-*goat IgG or Goat Anti*–*Mouse IgG) (Life Technologies) was added over 2 hrs at RT in the dark in PBS and BSA with the 1:500 concentration.

UCB and bone marrow cells-grown on polylysine (Sigma-Aldrich)-coated coverslips-were fixed over 10 min at room temperature (RT) in 2% paraformaldehyde, washed in PBS and incubated overnight in a humidified chamber at RT. Cells were then permeabilized over 10 min in 0.2% Triton, washed twice in PBS, and blocked over 1 hr using 150 ml of PBS and 10% Goat Serum (Life Technologies). Cells were stained overnight at 4 °C in primary antibody solution in PBS and goat serum with the concentrations as described above. After cells were washed twice in PBS, secondary antibody solution (goat anti-rabbit IgG or donkey anti*-*goat IgG or goat anti–mouse IgG) was added over 2 hrs at RT in the dark in PBS and goat serum with the 1:500 concentration.

Slides were visualized under widefield microscope. All microscopy images were captured using 100X oil-immersion objective and images were processed with Volocity software. Analysis was performed by counting the number of foci in 100 nuclei of each slide. Results were expressed as the average number of foci in each cell. For DNA damage distribution analysis, 100 cells were counted and cells were divided according to the number of foci per cells. Three slides per condition were analysed.

### Alkaline comet assay

The alkaline comet assay was performed according to previous protocols^56,57^. Cells with or without H_2_O_2_ treatment, were suspended in low melting point agarose in PBS, pH 7.4, at 37 °C and pipetted onto a frosted glass microscope slide pre-coated with 3 layers of agarose. The agarose was allowed to set at RT for 30 min and the slide immersed in lysis solution (2.5 M NaCl, 100 mM Na EDTA, 10 mM Tris, NaOH to pH 10.0, and 1% Triton X-100) at 4 °C for 1 h to remove cellular proteins. Slides were then placed in a single row in a 260 mm wide electrophoresis tank containing 0.3 M NaOH and 1 mM Na EDTA for 40 min, before electrophoresis at 40 V, 300 mA, for 30 min at an ambient temperature of 4 °C (the temperature of the running buffer not exceeding 15 °C). The slides were then washed 3 times for 5 min each dH_2_O, pH 7.5, at 4 °C before staining with 5 pg/ml ethidium bromide. Electrophoresis was performed at 4 °C in light-controlled conditions. A total of 300 cells with wells set up in triplicate for each treatment were scored at 400X magnification using a fluorescence microscope (Olympus BX-50) with an excitation filter of 515–560 nm and barrier filter of 590 nm and image analysis software (COMET III, Perceptive Instruments). DNA damage was evaluated by the tail moment (product of comet length and tail intensity). Fibroblasts were plated at 5 × 10^4^ per well into 12-well plates (Orange Scientific) and on round 18 mm glass coverslips for γ-H2AX assay, fixed in 4% formaldehyde and immunostained with polyclonal antibody to histone H2AX and donkey anti-rabbit IgG, secondary antibody. One hundred cells per well were scored in triplicate using an Olympus BX-41 microscope with image analysis software. The mean tail moment value is the mean of the comet tail of 100 cells. The average of the means of the 3 replicates is displayed. UCB cells were cultured in suspension in 12-well pates at 5 × 10^4^–1 × 10^6^ cells per well.

### BeWo barrier integrity measurement

Barrier integrity was checked using trans-epithelial electrical resistance (TEER) measurements and FITC- dextrans memberane permeability assay according to previous methods (77).

### Image stream

The Image Stream IS100 (Merck) equipped with 3 lasers (violet 405 nm, blue 488 nm and red 658 nm) and halogen lamp was used at the Nuffield Department of Medicine, University of Oxford. Antibody titration occurred prior to sample staining for all antibodies. UCB-MNCs were exposed indirectly to 30 μM HQ and BQ across bilayered cell barriers of BeWo cell line for 24 hours. Positive controls and single-stained ɣ-H2AX controls were subjected to 2 Gy X-ray radiation (Nuffield Department of Clinical Laboratory Sciences, John Radcliffe hospital, Oxford) 30 minutes prior to cell staining. Cells were washed in FC buffer (Hanks + 2% HAS) prior to staining. Staining occurred in 96 well round bottom plates (Corning) and centrifugation occurred at a speed of 500 × *g* for 5 minutes at 4 °C. Cells were washed and stained with combinations of the following cell surface antibodies: anti-human CD34-PE, anti-human CD38-APC/FITC (BD), anti-human CD3-APC, anti-human CD19-PE (eBioscience, Loughborough, UK), anti-human lineage cocktail-PB (CD3, CD14, CD16, CD19, CD20, CD56) (Biolegend, London, UK), for 25 min at RT on a shaker. Foxp3 transcription factor staining buffer kit was used (eBioscience). Cells were washed twice in FC buffer, fixed in 100 μL fixation buffer (eBioscience) for 15 minutes at room temperature on a shaker, washed twice in permeabilisation buffer and incubated with anti-human γ-H2AX-FITC (eBioscience) antibody for 20 minutes at RT on a shaker. After washing twice in FC buffer, cells were then re-suspendeded in 70 μL FC buffer and transferred to a 500 μL Eppendorf tube and kept in the dark until flow cytometric analysis. A minimum of 10^4^ focused live cells were collected per sample. Data was analysed using Amnis software (IDEAS). Single stained samples were used to create a compensation matrix. A data acquisition template was created and applied to each sample. This was firstly to identify live, in focus single cells based on autofluorescence in all channels, cell morphology (brightfield channel) and FSC-SSC parameters. Cell populations of interest were then identified by fluorescence intensity and relative quantification of y-H2AX calculated by utilising the IDEAS spot count analysis feature to determine the number of γ-H2AX foci/cell.

### Flow cytometry and cell sorting

A MACSQuant Analyser 10 (Miltenyi Biotec) or a LSR Fortessa (BD BD Biosciences) were used for flow cytometric analysis. Antibody labelling was carried out for 30–45 minutes at 4 °C. Propridium iodide (PI, BD Biosciences) or SYTOX Dead Cell Stains (Life Technologies) was added as a viability dye prior to analysis. Compensation was performed with single antibody-stained beads and for each antibody, an isotype control and a Fluorescence-Minus-One control was prepared in the same way as the samples. Data was analysed using FlowJo software (V10.0.07).

Cell sorting was performed on a BD Influx high speed machine or an ARIA cell sorter. Human MNCs were washed in FACS buffer (Pre-Sort Buffer (BD Biosciences) supplemented with 100 ug/ml DNase-II (Sigma-Aldrich) twice and stained with CD34-APC (eBioscience). A minimum of 10^5^ unsorted cells and CD34^−^ cells and between 5 × 10^2^ and 3 × 10^5^ CD34^+^ cells were collected per sample. Murine myeloid bone marrow cells were sorted on the basis of Gr1 and CD11b expression and lymphoid cells on the basis of CD3 and B220 expression. For the sorting of murine HSCs, red cell lysis buffer (STEMCELL Technologies) treated cells were first lineage-depleted using a Mouse Hematopoietic Progenitor Cell Enrichment Kit (STEMCELL Technologies). For further purification, a lineage cocktail (CD3e-APC, Ter119-APC, F4/80-APC, Nk1.1-APC, Gr1-APC, CD11b-APC, B220-APC, CD19-APC) was included in the antibody panel. The cells were further stained with Flk2-PE, Sca1-PB, ckit-APCeF780, CD48-FITC and CD150-PECy7 and Lin^−^/Sca1^+^/ckit^+^/Flk2^−^/CD48^−^/CD150^+^ HSCs were sorted.

### Cytogenetics

Metaphase studies, using an equal volume and dilution of each cell suspension, were carried out on SuperFrost slides fixed in Carnoy’s fixative (3:1 methanol: acetic acid). Slides were stained with 10% GIEMSA (Thermo Scientific) in dH_2_O for 15 min at room temperature. Numerical and structural chromosomal aberrations were scored in 100 metaphase spreads per experiment using a brightfield microscope DMLB2 (Leica) equiped with Colour DCF 450 C camera (Leica) or a fluororescent microscope A1AX10 (Zeiss) equipped with monochrome Retiga 300 camera (Q-imaging) using a 100x oil immersion lens. Images were scored using Image-J software. A mitotic rate was calculated as the number of mataphases per slide

### *In vitro*

All indirect exposures of cells to chemicals were made across bilayered cell barriers of the human trophoblast choriocarcinoma-derived BeWo cell line. BeWo barriers were grown on transwell inserts (pore size, 0.4 mm). Chemicals (HQ + BQ, N-Acetyl-L-cysteine, hexavalent Chromium ions, PolyI:C, LPS, Epicatechin, quercetin, etoposide, pyrethroid standard mixture, piperonyl butoxide) were placed in the top chamber. BeWo barriers were grown for up to 7 days to create predominantly bilayered barriers. Media with no chemical additions were used as controls. Primary BJ human fibroblasts and umbilical cord blood (UCB) cells were exposed to the condition media collected from the bottom of insert plates. In one experiment the cells were exposed to conditioned media with N-Acetyl-L-cysteine.

### *In vivo*

All animal experiments were performed according to institutional guidelines, UK Home Office approved project licence and a local University of Edinburgh Ethical Committee. The specifics of the experiments were further approved by the University of Edinburgh Veterinary Scientific Services. Animals were maintained in 12 h light/dark cycles with free access to food and water. Female C57BL/6 mice were mated with male C57BL/6 mice overnight and pregnant females were designated day 0.5 post-coitum (dpc) by the presence of a vaginal plug the next morning. HQ was administered orally by gavage (50 mg/kg body weight in H_2_O) at a volume of 10 ml/kg on GD 12 and controls were treated with H_2_O only. γ-PGAPhe nanoparticles (70) bound to MitoQ (56) were resuspended in 100 µl PBS (334.5 ng/ml) to give a final and equivalent concentration of 0.5 µM of MitoQ in the peripheral blood and were administered intravenously into the tail vein in a single injection in one population of pregnant mice at GD 12. Controls were injected with PBS only. For each experimental group, four mice were analysed. Total bone marrow samples from newborn foetuses were cultured in RPMI medium supplemented with 10% FBS, 1% penicillin- streptomycin and 1% L-glutamine at 5% O_2_, 5% CO_2_, 37 °C. Some newborns were left to be weaned and the immunostimulant polyI:C was injected intraperitoneally (IP) at 4-weeks of age (5 mg/kg) as an infective mimic to simulate viral infections. Total bone marrow (BM) of the newborn mice was analysed for DNA damage. All bone marrow cells in this study were extracted from the femurs and tibiae of the mice.

### *Ex vivo*

To investigate the effect of radiation-induced bystander mechanism, adult female C57BL/6J mice at GD12 were exposed to a whole body exposure 100 mGy or 1 Gy X-irradiation (AGO, United Kingdom, dose rate 0.5 Gy/min (250 kVp and 13 mA)). All animals were bred and handled according to the UK Animals (Scientific Procedures) Act, 1986, Amendment Regulations 2012, and animal experimental protocols were reviewed and approved by the PHE CRCE Ethics Committee and the Home Office. The placentae were removed 4 hrs post-irradiation and cultured in RPMI medium supplemented with 10% FBS, 1% penicillin- streptomycin and 1% L-glutamine with 3 conditions: no drug additive, with MitoQ bound to NP (MQNP) (334.5 ng/ml nanoparticles, 0.5 µM MitoQ) and with blank NP (BNP). Cultured explants were maintained in 5% O_2_ at 37 °C for 24 h. Total BM from age-matched female C57Bl/6J was exposed to conditioned media.

To investigate the effect of indirect (transplacental) exposure of mouse BM cells to HQ, total BM from age-matched female C57BL/6 mice was exposed to the conditioned media obtained from the explant placenta removed from mice at birth (GD18) that had been exposed to hydroquinone at GD12, 6 days earlier (see *in vivo*). The BM cells were cultured in RPMI medium in 5% O_2_ at 37 °C for 24 h.

### Statistics

Statistical analysis was performed using Prism 5.0 (GraphPad Software Inc.) and Microsoft Excel. Data are presented as mean ± SD (standard deviation). Where appropriate, parametric two-tailed student’s *t*-tests were applied to compare for significant differences between groups. When comparing more than 2 parameters, two-way ANOVAs (multiple independent groups) followed by Tukey post hoc testing (when *p* < 0.05) were performed.

## Supplementary information

Supplementary Information
