# Supplementary material for: DNA damage signalling from the placenta to foetal blood as a potential mechanism for childhood leukaemia initiation
Source: Sci Rep. 2019 Mar 13;9:4370. doi: 10.1038/s41598-019-39552-0 (PMC6416312; doi:10.1038/s41598-019-39552-0)
Supplement: Supplementary file 1 — Supplementary Information [file 41598_2019_39552_MOESM1_ESM.pdf]

Supplementary

DNA damage signalling from the placenta to foetal blood as a potential mechanism for childhood leukaemia initiation

Authors

Els Mansell[1+], Nahid Zareian[1+], Camille Malouf[2], Chrysa Kapeni[2], Natalie Brown[3], Christophe Badie[3], Duncan Baird[4], Jon Lane[5], Katrin Ottersbach[2], Allison Blair[5] and C. Patrick Case [1*]

A

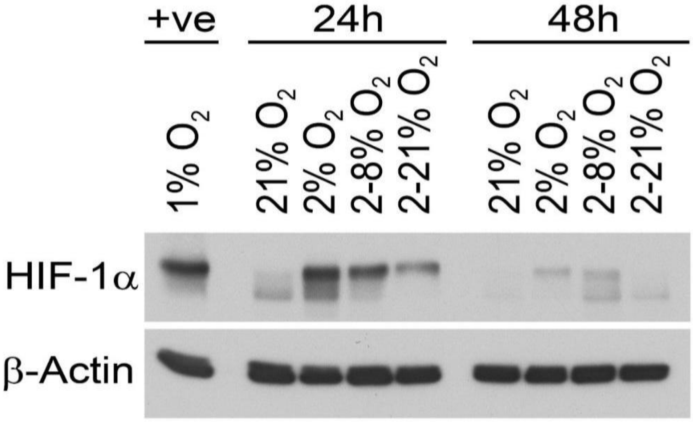

B

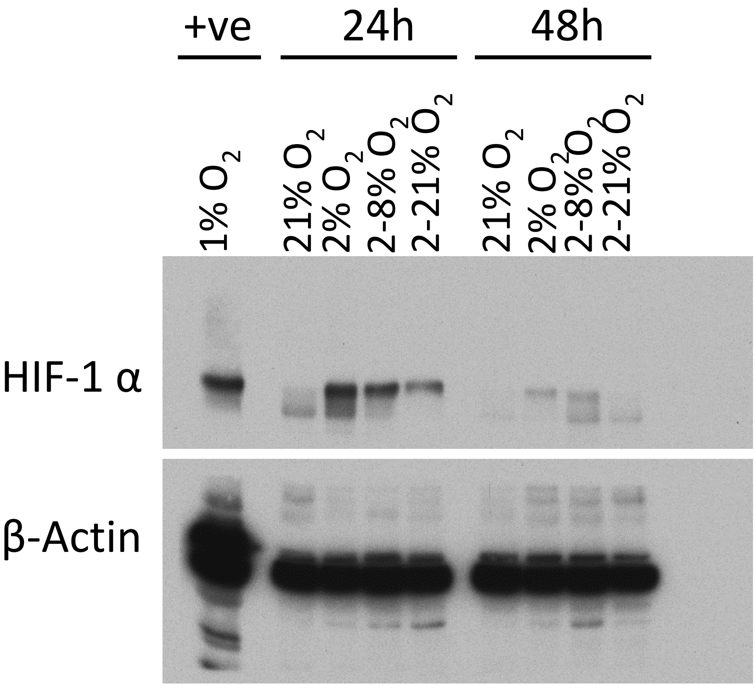

C

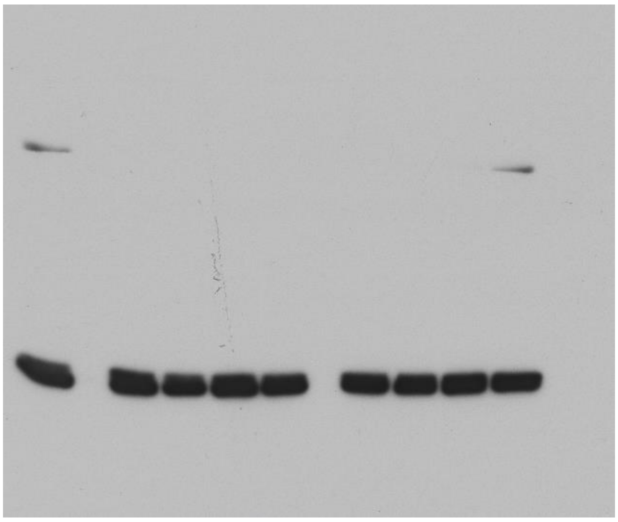

β-Actin

Figure S1- Western blot of hypoxia- inducible factor 1-alpha (HIF-1α) (92kDa) and loading control β-actin (42kDa) on protein lysates from BeWo cells exposed to various oxygen concentrations in the hypoxic chamber over a period of 48 hours. A) Bands are shown in the summary gel and the fuller length gel at **B)** higher and **C)** lower exposure after an exposure of the barriers to different levels of oxygen for 24 hours (21%, 2% O2), or hypoxia for 24 hours followed by reoxygenation for 24 hours (2-8%, 2-21% O2) in the summary. The positive control consisted of SW620 cells exposed to 1% O2 for 24 hours and the negative control consisted of BeWo cells grown at atmospheric (21%) oxygen.

A) BQ- Positive Control

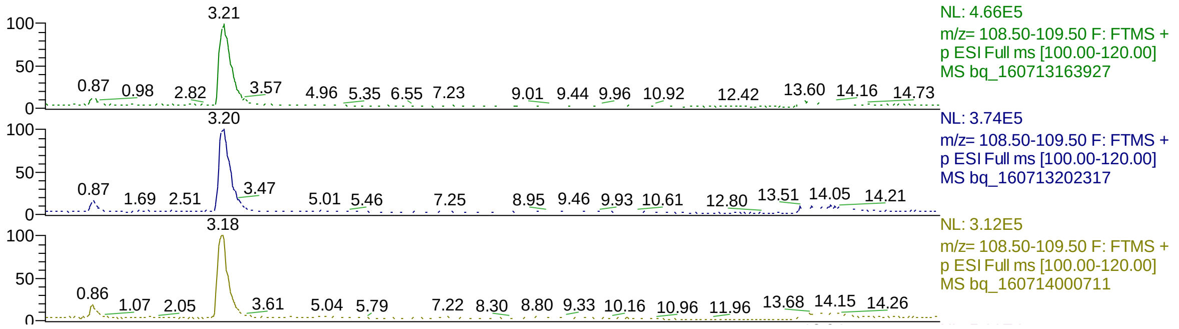

B) Conditioned Media from media only-exposed BeWo Barrier

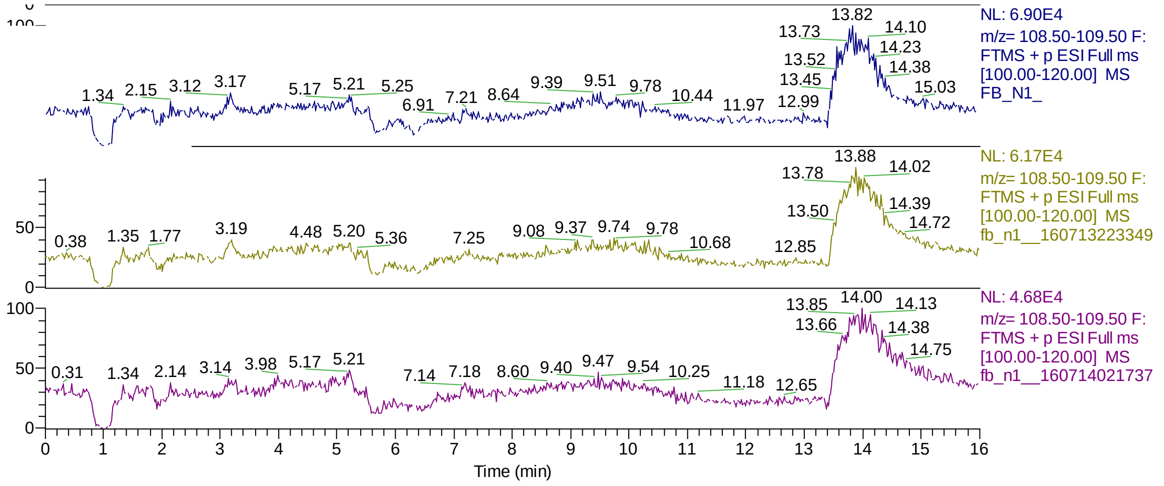

C) Conditioned Media from BQ+HQ-exposed BeWo Barrier

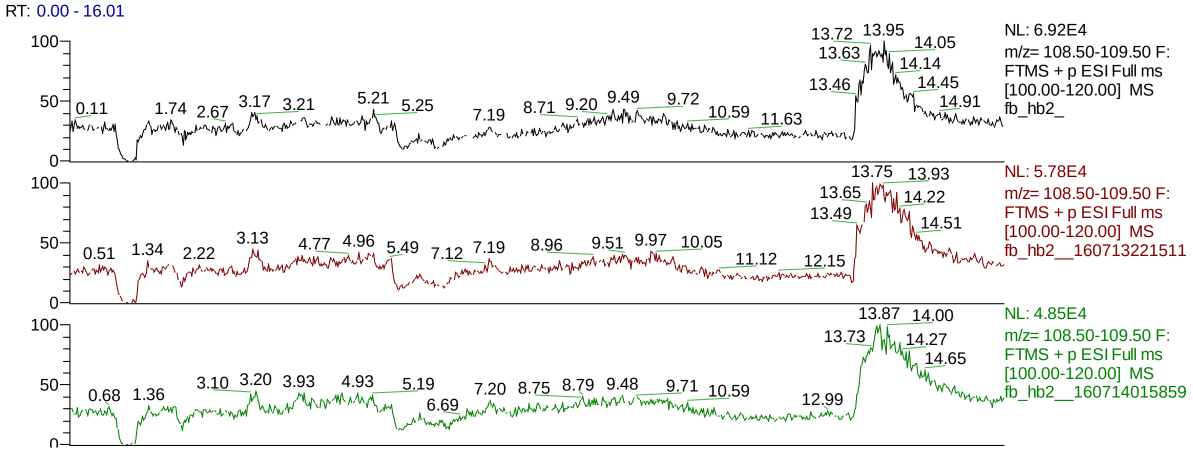

Figure S2- Selective ion monitoring (SIM) of benzoquinone (109.03Da). A) Positive control sample (standard) where BQ elutes at 3.2 minutes. **B**, **C)** BQ was not observed in the conditioned media of samples that were exposed to plain media or BQ through the BeWo barrier, respectively.

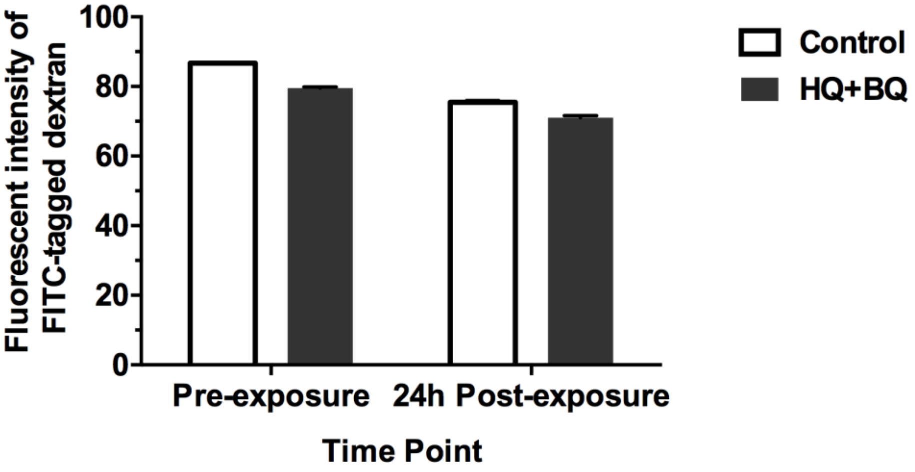

Figure S3- Measurement of BeWo barrier permeability after exposure to culture medium without (open histograms) or with (shaded histograms) 30μM benzoquinone and hydroquinone for 24 hours. Measurements show concentration of FITC- labelled dextrans in the media below the barrier after placing 0.5 ml of 10 μM of FITC-dextrans above the barrier for 24 hours. Error bars represent SD, n=3.

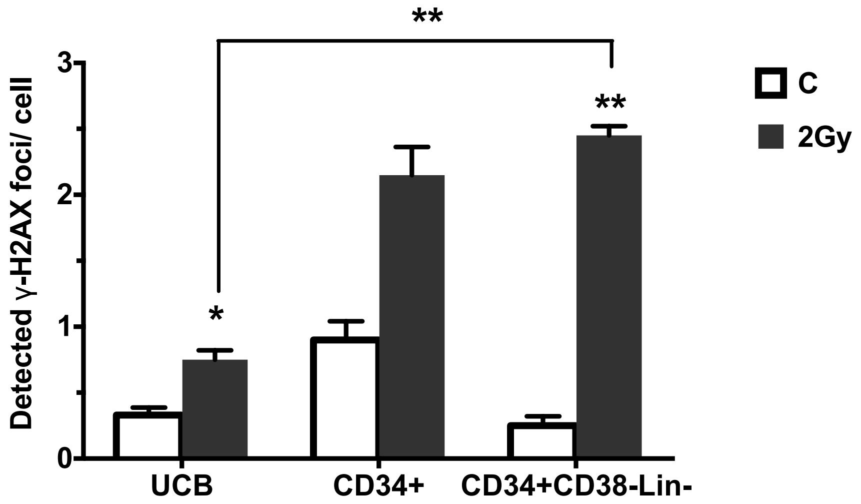

Figure S4- Image Stream counts of umbilical cord blood (UCB) cells exposed to X irradiation. Image Stream γ-H2AX counts of cord blood cells after normal culture conditions or exposure to 2Gy of X-irradiation. UCB represents the bulk of umbilical cord blood mononuclear cells, CD34+ cells represent bulk hematopoietic stem/progenitor cells, CD34+CD38-Lin- cells represent HSCs. Asterisks on solid column represent significant difference between control (C) and 2Gy condition in each group. The hairpin compares the 2Gy conditions. Error bars represent SD. n=3. *p<0.05, **p<0.01 as determined by unpaired two-tailed student's *t*-test.

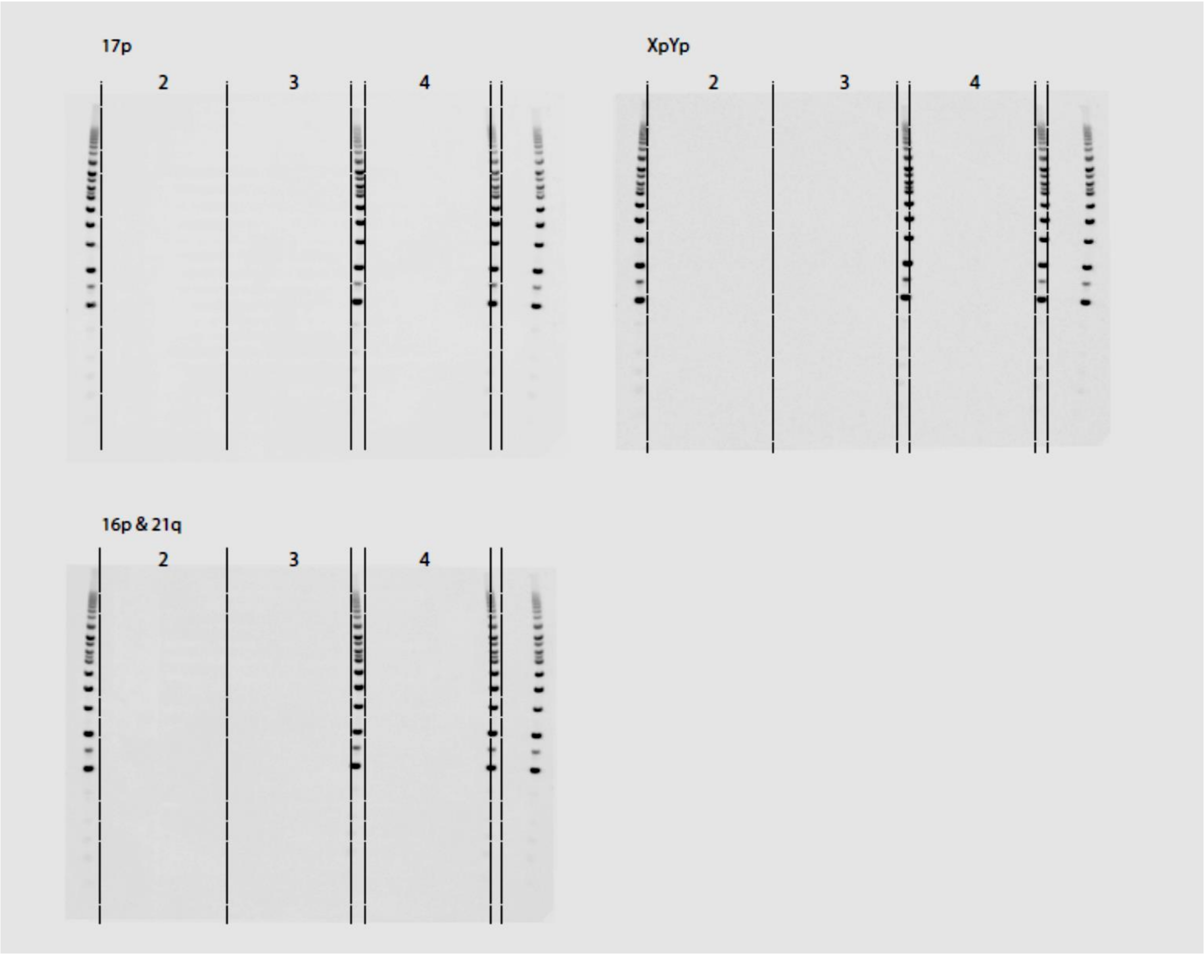

Figure S5- Telomere fusion PCR from cord blood. Results from a fusion gene PCR assay detecting 17p, XpYp and 16p & 21q telomeric fusion events. DNA samples are taken from CB mononuclear cells (MNCs) exposed to plain BeWo CM (2), or BeWo CM from 30uM BQ and HQ exposure (3) or 100 ug/ml pesticide exposure (4). Visible bands represent the DNA marker lanes. Visible bands in the lanes below numbers 2,3 and 4 would indicate presence of telomeric fusions.

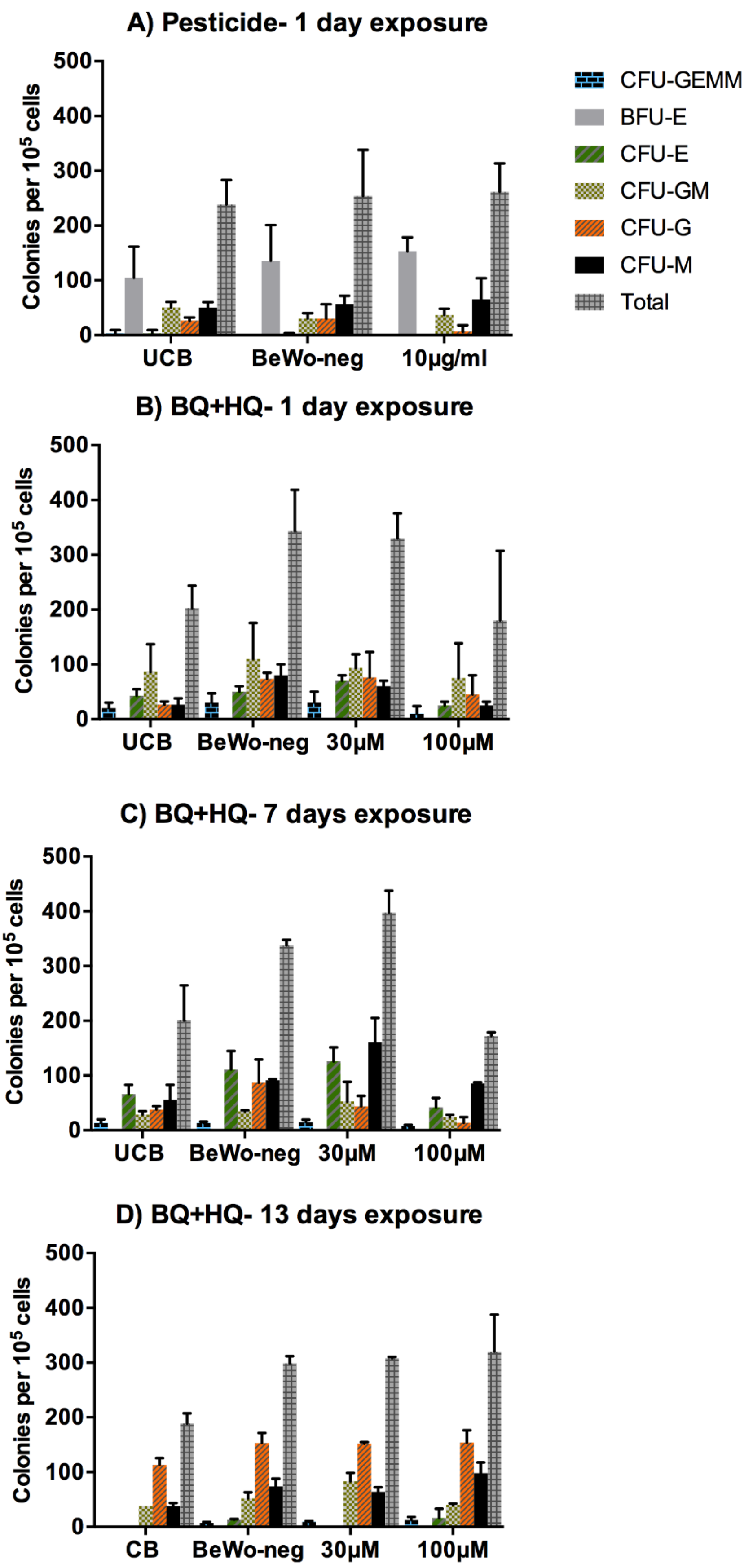
A) Pesticide- 1 day exposure
Colonies per 10^5 cells
500
400
300
200
100
0
UCB
BeWo-neg
10μg/ml
CFU-GEMM
BFU-E
CFU-E
CFU-GM
CFU-G
CFU-M
Total
B) BQ+HQ- 1 day exposure
Colonies per 10^5 cells
UCB
BeWo-neg
30μM
100μM
C) BQ+HQ- 7 days exposure
Colonies per 10^5 cells
UCB
BeWo-neg
30μM
100μM
D) BQ+HQ- 13 days exposure
Colonies per 10^5 cells
CB
BeWo-neg
30μM
100μM

Figure S6- CFU results from UCB MNCs after exposure to pesticide or benzene BeWo conditioned media (CM) at different time points. Results from CFU assays of UCB MNCs that were cultured in methylcellulose after normal culture conditions (UCB), exposure to BeWo CM from plain BeWo barriers (Bewo neg) or **A)** BeWo CM from 10 μg/ml pesticides for 1 day; **B, C, D)** BeWo CM from 30 μM and 100 μM BQ+HQ for 1, 7 and 13 days' exposure, respectively. Bars represent colonies per 10^5 cells. Error bars represent SD, n=3.

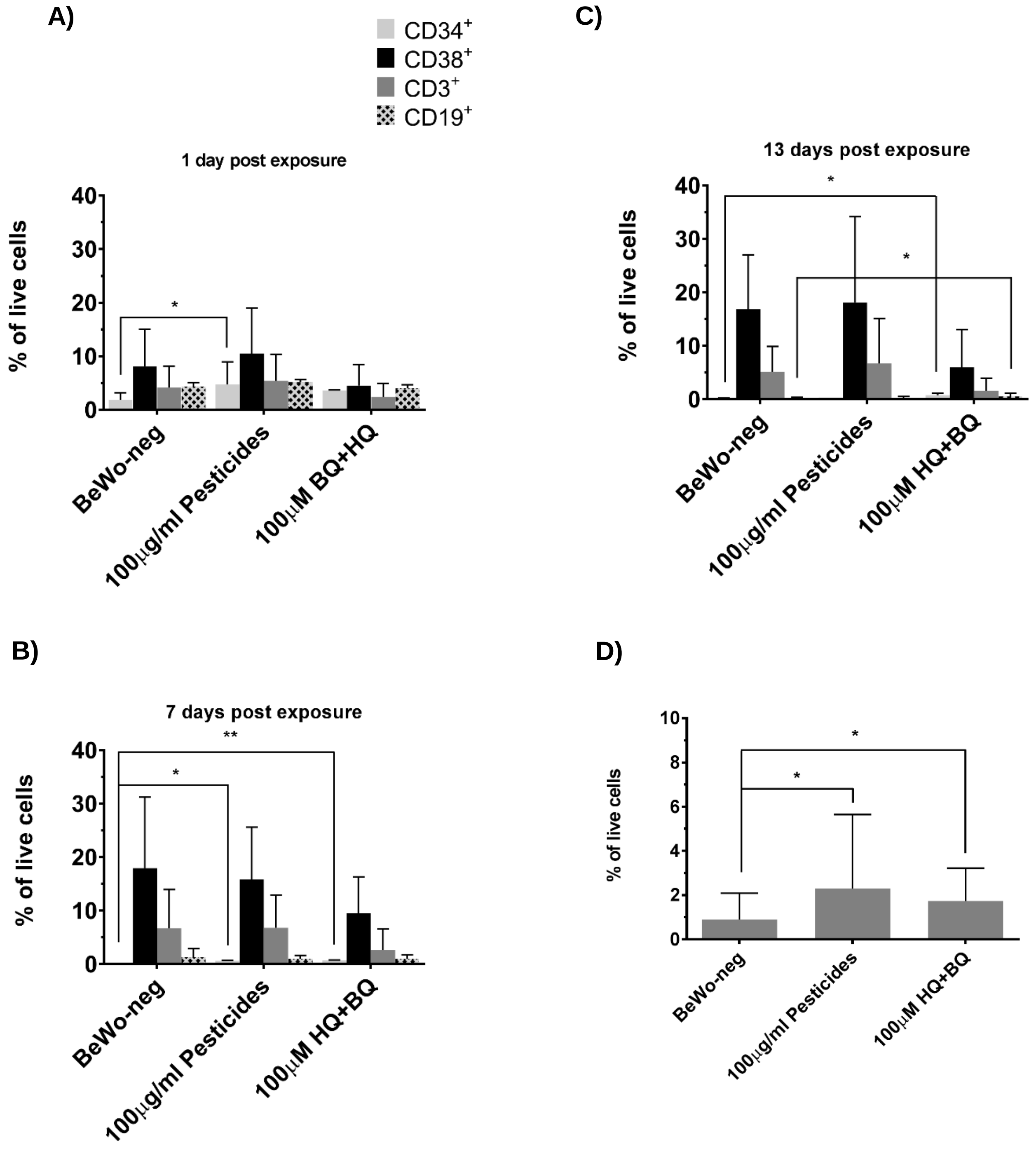

Figure S7- Flow cytometric population analysis after exposure to pesticide and BQ+HQ BeWo conditioned media (CM) at different time points. Results from flow cytometric lineage assessment of the UCB MNC fraction, after exposure to BeWo CM from plain BeWo barriers or BeWo CM from 100µg/ml pesticide exposure or 100µM BQ+HQ after **A)** one day; **B)** 7 days and **C)** 13 days in culture. **D)** Mean values for CD34+ cells when combining values for 1, 7, 13 days. Bars represent percentage of live cells. Error bars represent SD. n=3. * $p<0.05$, ** $p<0.01$ as determined by unpaired two-tailed student's *t*-test.

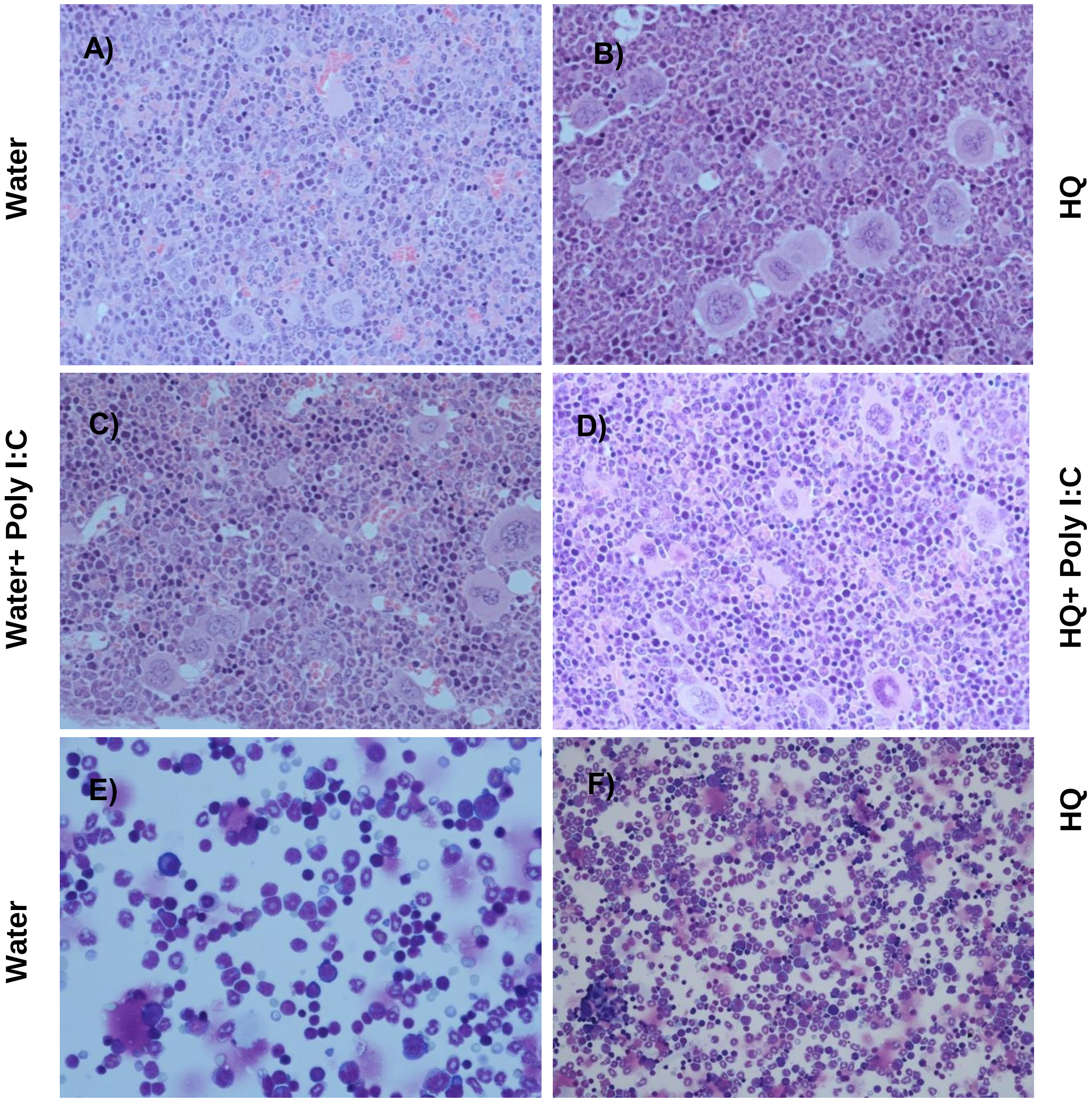

Figure S8- Photomicrographs of HE stained sections and aspirates of femoral bone marrow from 4 week+ 4 day old mice after intrauterine exposure to HQ with and without postnatal exposure to PolyI:C 4 weeks after birth. A-D) HE stained sections of femoral bone marrow of the offspring (Magnification: x400) after maternal

exposure to **A)** water (control); **B)** HQ; **C)** water (maternal exposure) followed by PolyI:C (exposure of offspring); and **D)** HQ (maternal exposure) followed by PolyI:C (exposure of offspring). **E, F)** Giemsa stained bone marrow aspirates of the same bone marrows after maternal exposure to **E)** water (control) (x400); or **F)** (hydroquinone) x200).
